# The molecular mechanism of macrophage-adipocyte crosstalk in maintaining energy homeostasis

**DOI:** 10.3389/fimmu.2024.1378202

**Published:** 2024-04-08

**Authors:** Yudie Zhang, Bin Zhang, Xiaobo Sun

**Affiliations:** ^1^ Institute of Medicinal Plant Development, Peking Union Medical College and Chinese Academy of Medical Sciences, Beijing, China; ^2^ Guizhou University of Traditional Chinese Medicine, Guiyang, China; ^3^ Key Laboratory of Bioactive Substances and Resources Utilization of Chinese Herbal Medicine, Ministry of Education, Beijing, China; ^4^ Beijing Key Laboratory of Innovative Drug Discovery of Traditional Chinese Medicine (Natural Medicine) and Translational Medicine, Beijing, China; ^5^ Key Laboratory of Efficacy Evaluation of Chinese Medicine Against Glyeolipid Metabolism Disorder Disease, State Administration of Traditional Chinese Medicine, Beijing, China

**Keywords:** macrophage polarization, mitochondrial function, inflammation, energy metabolism, obesity, brown adipose tissue, fat

## Abstract

Interactions between macrophages and adipocytes in adipose tissue are critical for the regulation of energy metabolism and obesity. Macrophage polarization induced by cold or other stimulations can drive metabolic reprogramming of adipocytes, browning, and thermogenesis. Accordingly, investigating the roles of macrophages and adipocytes in the maintenance of energy homeostasis is critical for the development of novel therapeutic approaches specifically targeting macrophages in metabolic disorders such as obesity. Current review outlines macrophage polarization not only regulates the release of central nervous system and inflammatory factors, but controls mitochondrial function, and other factor that induce metabolic reprogramming of adipocytes and maintain energy homeostasis. We also emphasized on how the adipocytes conversely motivate the polarization of macrophage. Exploring the interactions between adipocytes and macrophages may provide new therapeutic strategies for the management of obesity-related metabolic diseases.

## 1 Introduction

Obesity, characterized as excess adipose tissue accumulation, has become a serious global health issue because of its induced risk of metabolic diseases, immune dysfunctions, cardiovascular diseases, and cancers (1–3). Proverbially, adipose tissues can be divided into two types: white adipose tissue (WAT) mainly for the storage of fat, and brown adipose tissue (BAT) for the generation of energy by nonshivering thermogenesis (4–6). Increasing evidence also indicates that white adipocytes can be differentiate into beige adipocytes containing a small number of mitochondria (7).

Numerous researches have reported that adipose tissues are endocrine organs, and their homeostasis is controlled by internal immune cells, including adipose tissue macrophages (ATMs) (8–10). Both macrophages’ recruitment and polarization can regulate the microenvironment in the adipose tissues (11–14). It has been clarified that the main sources of ATMs are monocyte-derived recruited macrophages as well as tissue-resident macrophages. In obesity, the adipocytes and stromal vascular fraction can produce some monocyte chemoattractant proteins, such as monocyte chemoattractant protein-1(MCP1), leading to the enhancement of macrophage infiltration into the adipose tissues (15–17). Even worse, ATMs in the obese are found to polarize to a pro-inflammatory phenotype, subsequently leading to insulin resistance and other metabolic diseases. Conversely, tissue-resident macrophages could exhibit the phenotype of anti-inflammation and facilitate the development of brown or beige adipose tissues. Thus, understanding how adipose tissues regulate macrophage recruitment and polarization is of great importance for the prevention of obesity.

Classically, ATMs are broadly classified into two types: activated M1 macrophages and alternatively-activated M2 macrophages (10). Under different microenvironments, AMTs are rapidly capable of adapting through peripheral cell recruitment as well as proliferation. In lean adipose tissue (AT), the macrophages mainly exist as M2 subtypes to contribute to AT repair and eventually to maintain the homeostatic environment of AT (18–20). However, this balance will be disrupted by obesity, in which the numbers of ATMs are further increased by 5–10 fold (21, 22). Accompanying the metabolic abnormality, the malleable macrophages quickly alter their phenotype and function (23), to subsequently secreting many pro-inflammatory cytokines (e.g., IL-1β and TNF-α) that could influence adipocyte remodeling and impair insulin sensitivity (24). Although some reviews have reported the key roles of macrophages in the AT, these summaries predominately concentrate on the origin of macrophages and the mechanisms that are related to polarization. Thus, a comprehensive review of the crosstalk between macrophages and adipocytes remains limited and urgently needed.

In this article, we comprehensively summarized the interplay between macrophages and adipocytes in controlling immunological regulation and energy metabolism regulation, with a focus on elucidating the essential regulatory factors governing macrophage-adipocyte crosstalk as well as the underlying molecular mechanism, providing effective strategies for the prevention of obesity-related diseases such as insulin resistance (25), atherosclerosis (26), type 2 diabetes and nonalcoholic fatty liver diseases (NAFLD) (27, 28).

## 2 Adipose tissue performs a crucial role in preserving energy homeostasis

Adipose tissue is a highly heterogeneous, complex, and mini-endocrine organ (29, 30). As early as 1994, the subsequent identification and characterization of leptin established the status of adipose tissue as an endocrine organ (31). Adipose tissue not only sends out signals but also expresses many receptors that enable it to respond to signals from the hormonal system and the central nervous system (CNS) (29). Therefore, in addition to storing and releasing energy, adipose tissue also regulates metabolism, enabling it to communicate with distant organs, including the central nervous system, forming a large interaction network that engages adipose tissue in coordinating various biological processes, including energy metabolism, neuroendocrine function, and immune function.

**Figure 1:**
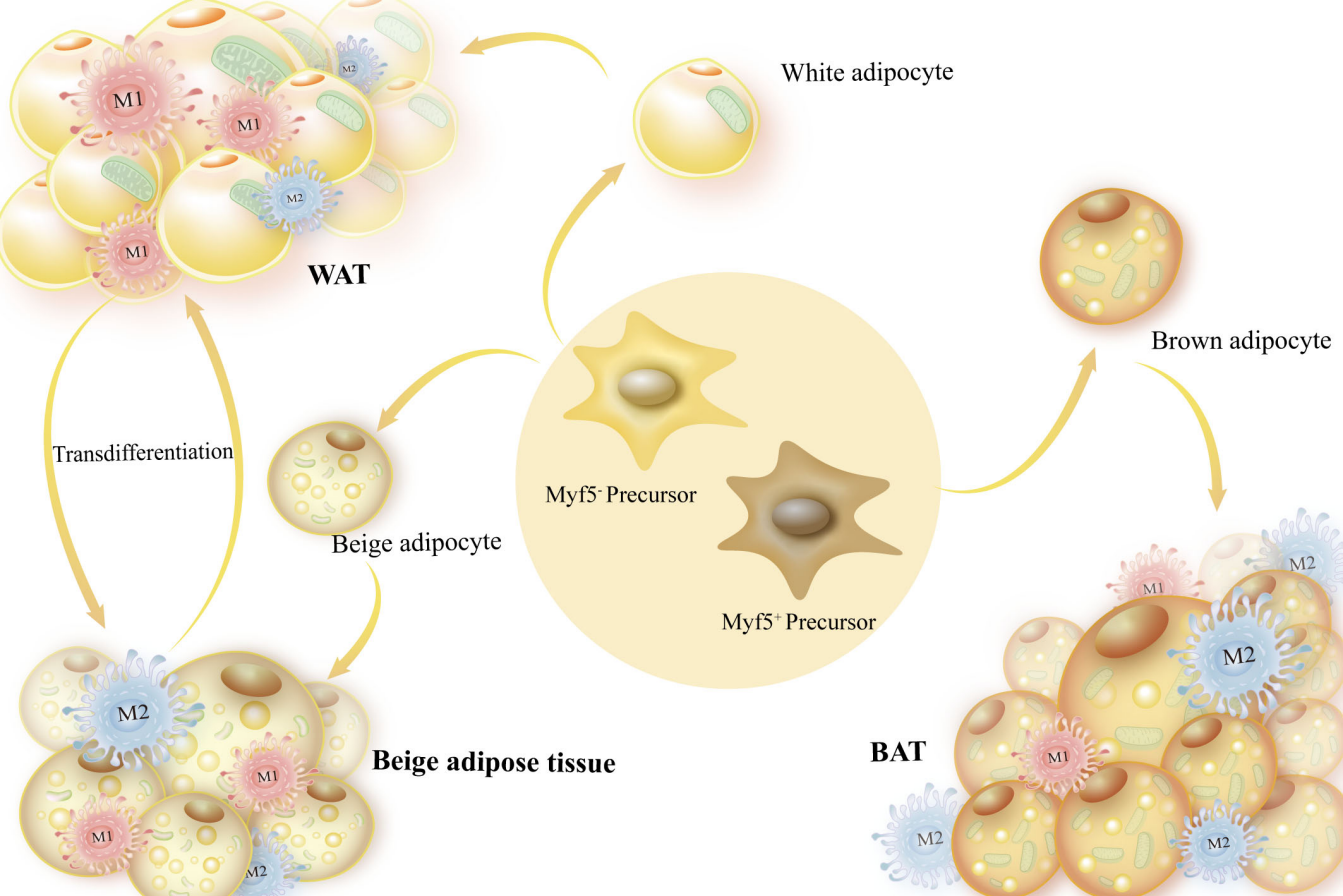
Sources of adipocytes and adipose tissue categorization. Myf5- precursors, or white adipose tissue, are the source of white and beige adipocytes, which are mostly employed for energy storage. Brown adipocytes are produced by Myf5+ precursors and have a high number of mitochondria, which are necessary for producing heat.

The origin of white adipocytes and brown adipocytes is different. White adipocytes and beige adipocytes with a limited number of mitochondria originate from Myf5^-^ precursor and can occasionally differentiate into each other (32). Brown adipocytes source from Myf5^+^ precursor, which contain a large number of mitochondria and are essential for thermogenesis. Precursor are malleable and can be stimulated by both internal and external tissue environments. They can be subjected to systematic regulation of immune cells, different inflammatory factors, and the extracellular matrix to determine their own differentiation potential, which is important for maintaining the body’s metabolic homeostasis (6, 33–35) (**Figure 1**). The functional characteristics of various forms of adipose tissue in energy metabolism are the main topic of this section.

### 2.1 WAT’s significance for energy storage

The human body contains a large amount of WAT, which is primarily found in the subcutaneous regions of the buttocks, deep and shallow abdomen, and visceral area, where it is located beneath the skin. WAT serves as a buffer against external mechanical stress, an insulator against heat loss, and a barrier against skin infections. Vital organs in the peritoneum and ribs of the body are encased in visceral white fat (36).

Triglycerides, which WAT stores as energy, are also a source of energy, which is why they are important for preserving energy homeostasis. Excess fats are stored as triglycerides in WAT when the body uses too much energy. This keeps lipids from building up in other organs, such as the liver, where they could impair normal bodily functions or cause harmful metabolic issues. Moreover, WAT serves as a source of energy in the presence of starvation, malnutrition, and other conditions. The body responds to these stimuli by regulating catecholamines and sympathetic nervous system signals. Adipose triglyceride lipase (ATGL), hormone-sensitive lipase (HSL), and monoglyceride lipase (MG) work together to hydrolyze triacylglycerol in adipose tissue, which is then released into the bloodstream. Part of the fatty acids are oxidized by beta oxidation and then the products go into the tricarboxylic acid cycle to produce ATP (5, 37).

Additionally, when WAT browning occurs under certain stimulations, it would become an organ for heat production. It was discovered that inhibition of notch receptor-1 (Notch-1) signaling, deletion of the immediate early response gene X-1 (IEX-1), and activation of adenosine 5’-monophosphate-activated protein kinase (AMPK) and p38 mitogen-activated protein kinase (p38 MAPK) signaling, all promoted WAT browning (38–40). These signaling pathways have been reported to be linked to immunological responses and macrophage polarization, and offer prospective targets for the treatment of metabolic illnesses, such as obesity.

### 2.2 Beige adipose tissue’s function in maintaining energy homeostasis

Beige adipocytes can be formed through progenitor cell differentiation as well as through the transformation of mature white adipocytes through the activation (or reactivation) of thermogenic processes and the proliferation of mature beige adipocytes (7, 41, 42).

Beige adipocytes can be distinguished from white adipocytes due to their expression of uncoupling protein 1 (UCP1) and low mitochondrial count. UCP1, as an inner mitochondrial membrane transporter protein, is able to reduce the pH gradient formed by oxidative phosphorylation so that ATP synthesis is uncoupled from oxidative phosphorylation and energy is released as heat (43). WAT will turn beige and increase energy expenditure in the presence of a cold or increased energy demand, hence delaying the onset of metabolic illness and obesity (6). Without UCP1, beige fat uses glucose more efficiently by increasing the activity of pyruvate dehydrogenase, glycolysis, and tricarboxylic acid metabolism. This leads to ATP-dependent thermogenesis through the Sarco/endoplasmic reticulum (ER) Ca ^2+^ -ATPase 2b (SERCA2b) pathway (44).

### 2.3 BAT contributes to energy metabolism

The crucial and unique role that brown adipocytes play in energy metabolism is determined by their structural differences from white adipocytes. The inner mitochondrial membrane of brown adipocytes contains the BAT-specific protein UCP1, along with a profusion of mitochondria and a few tiny lipid droplets. BAT uses UCP1 to react to cold stimuli, which also causes it to boost the oxidation of glucose and free fatty acids, maintain high levels of mitochondrial and uncoupled respiration, control non-trembling thermogenesis, and regulate systemic energy balance (38). BAT is mainly found in small mammals and newborns, while it is less distributed in adults (40, 45). Generally, brown adipocytes use the β-adrenergic receptor (β-AR)-dependent signaling pathway and mitochondrial UCP1 to convert energy into heat. If UCP1 is not present, the triglyceride/fatty acid cycle plays a significant role in the heat production of brown adipocytes (46) (47). Considering these two thermogenic pathways, this section will explain how BAT controls energy metabolism, homeostasis, and consumption of energy.

#### 2.3.1 UCP1 controls non-trembling thermogenesis

**Figure 2:**
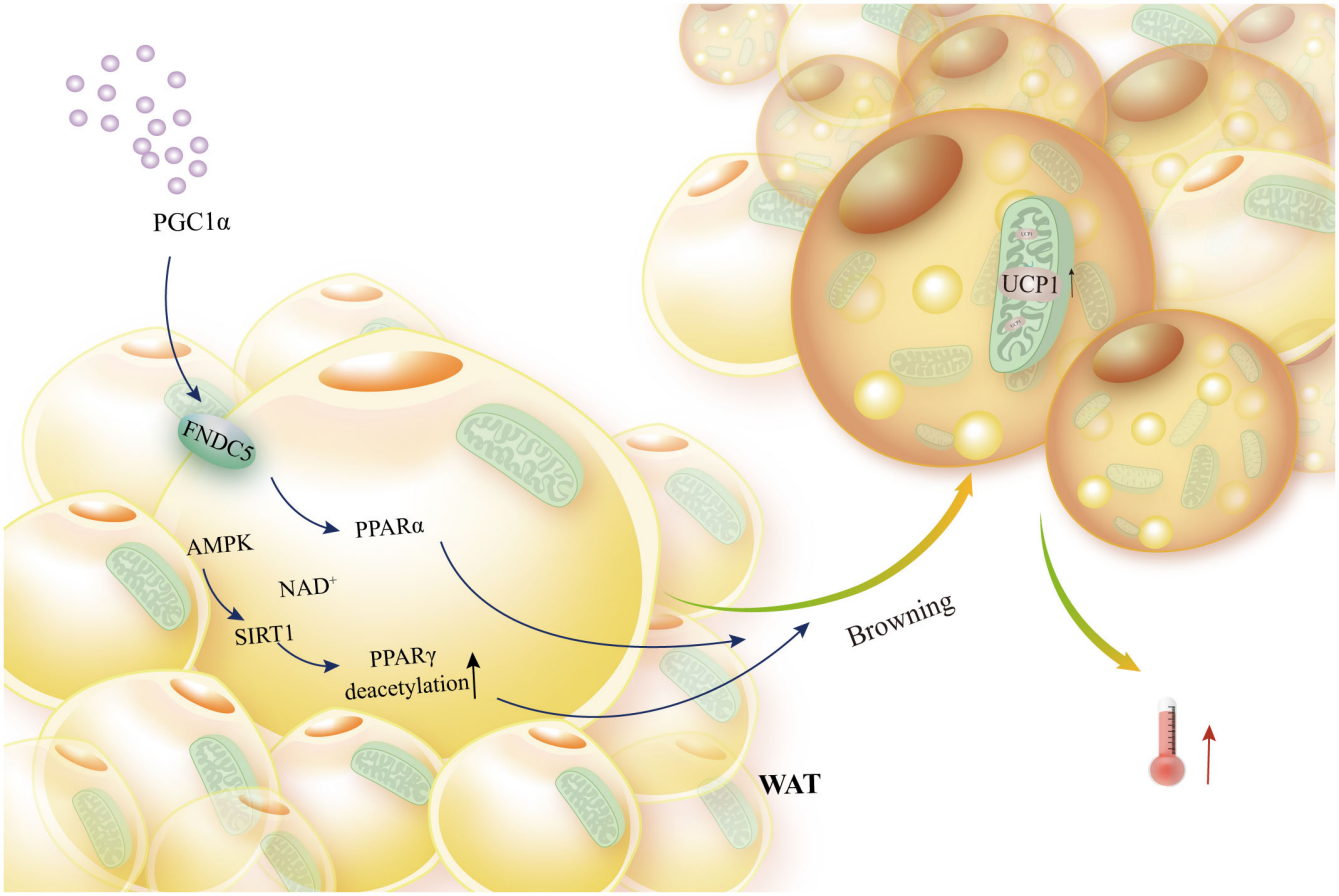
Brown adipocytes’ browning of white fat and UCP1 activation. External PGC1α In order to activate PPARα, FNDC5 expression must be boosted. Similarly, the AMPK-SIRT1 axis must activate PPARγ in order to induce WAT browning, elevated UCP1 expression, and thermogenesis.

BAT plays a key role in energy expenditure through UCP1-mediated thermogenesis. Hence, a crucial element in brown fat thermogenesis is UCP1 expression and the downstream signals it generates. When peroxisome proliferator-activated receptor gamma coactivator 1 alpha (PGC1α) is expressed, it increases the expression of fibronectin type III domain-containing 5 (FNDC5), which in turn promotes UCP1 expression and brown adipose-like growth. This improves glucose homeostasis and obesity by increasing energy expenditure (48). Additionally, the energy metabolism sensing pathway’s AMPK/SIRT1 axis activation raises the deacetylation level of peroxisome proliferator-activated receptor gamma (PPARγ), which encourages the remodeling of adipose tissue, and increases the expression of the thermogenic protein UCP1, which encourages the remodeling of adipose tissue and thermogenesis (49, 50). Further, when long-chain fatty acids (LCFAs) are activated, UCP1 increases the conductance of the inner mitochondrial membrane (IMM), which produces heat in BAT mitochondria (51) (**Figure 2**).

The regulation of BAT and energy balance is significantly impacted by the succinylation of UCP1 and other mitochondrial proteins. UCP1 is succinylated and malonylated much more when BAT is specifically lacking in sirtiun 5 (mitochondrial desuccinase and desmalonylase). As a result, energy homeostasis is disturbed, and UCP1’s stability and activity are significantly decreased (52). On the other hand, when expressed in inguinal subcutaneous fat (SC) and epididymal fat (EP), mitochondrial heat shock protein 60 (Hsp60) promotes thermogenesis and triggers UCP1 production (53). The UCP1 transcriptional regulator enzyme influences BAT thermogenesis in a manner akin to that of mitochondrial proteins. A substantial decrease in UCP1 transcript expression results from a BAT-specific deletion of mettl3, which impacts BAT development, inhibits adaptive thermogenesis, and exacerbates obesity and systemic insulin resistance linked to a high-fat diet (54). Reactive oxygen species produced by thermogenic succinate dehydrogenase-mediated succinate oxidation have been demonstrated in several investigations to improve brown adipose tissue respiration *in vivo*, hence inducing UCP1-dependent thermogenesis and systemic energy expenditure (55, 56).

Activation of carbohydrate response element binding protein β (ChREBP-β) promotes the AKT2-ChREBP pathway in BAT and accelerates *de novo* lipogenesis (DNL) in adipocytes, thereby optimizing energy storage and calorie production (57). In addition, ChREBP BAT-specific knockout mice exhibits an evident decrease in the *de novo* adipogenic activity, and reduced expression of UCP1 after acute cold exposure but not chronic cold stimulation (58). This implies that the duration of cold stimulation is differential for ChREBP-mediated increases in UCP1, and further studies are needed to fully understand the effects of ChREBP and its isozymes on BAT-mediated thermogenesis. In addition, with the adrenergic stimulation, the mitochondrial calcium transporter (MCU) has been found to recruits UCP1 to form a thermoporter to enhance thermogenesis (59). Thus, the MCU would be a significant energy source for UCP1’s thermogenic process, which would be advantageous for the management of metabolic illnesses like obesity.

Unexpectedly, thermogenesis in brown fat also seems to be tightly correlated with inflammatory variables. By inhibiting the excessive recruitment of inflammatory cells into the WAT and promoting the BAT’s thermogenic activity, adipocyte C-X-C motif chemokine receptor 4 (CXCR4) prevents the development of obesity (60). In cold-stimulated mice, ablation of IL18r1 sustains body temperature by promoting fat browning with the ubiquitous expression of thermogenic genes and enhanced macrophage alternative activation (61). Lack of IL-6 improves glucose homeostasis and respiratory exchange rate, which in turn promotes BAT thermogenesis (13).

#### 2.3.2 Thermogenic activity mediated by fatty acid oxidation

Triglycerides in lipid droplets are broken down by cold stimulation into free fatty acids, which are oxidized by beta oxidation and the products enter the tricarboxylic acid cycle to produce ATP to maintain energy balance (62–65). Angiopoietin-like 4 (ANGPTL4) regulates lipoprotein lipase (LPL) activity, affects triglyceride and fatty acid hydrolysis, and regulates thermogenesis (66).

Weight reduction and improved insulin sensitivity are the outcomes of the lactate receptor carboxylic acid receptor 1 (HCAR1) expressed in BAT, which enhances glucose uptake, improves glucose homeostasis, and decreases lipolysis (67). In brown adipocytes, lipolysis, fatty acid oxidation, oxidative metabolism, and thermogenesis are significantly boosted by transcriptional regulators interacting with PHD zinc finger and/or bromodomain 2 (TRIP-Br2) deletion. One possible strategy to counteract obesity-induced BAT dysfunction is to inhibit TRIP-Br2 (68).

In conclusion, signaling pathways, including AMPK, p38 MAPK, etc., control the browning and thermogenesis of adipose tissue. The expression and activation of UCP1 and its downstream signaling are crucial to thermogenesis. It is noteworthy that the amount of inflammatory chemicals and the activation of macrophage replacement had an indirect effect on browning and thermogenesis in adipose tissue.

## 3 Macrophages indirectly regulate energy homeostasis

### 3.1 Macrophage polarization and function in metabolism

Immune cell function is largely influenced by their metabolic activity, so immune cells need to acquire specific metabolic adaptations to support their diverse immunological functions. Nobel laureate Elie Metchnikoff first hypothesized and highlighted macrophages as a crucial component of innate immunity at the end of the 19th century (69). Admittedly, Hematopoietic stem cells produce monocytes. As the precursor for macrophages, they can mature into circulatory monocytes when entering the peripheral circulation. After that, these monocytes leave the circulation and settle into the tissue, where they mature into tissue-specific macrophages. Different tissues refer to resident macrophages by different names: adipose tissue macrophages, kupffer cells in the liver, and microglial cells in the central nervous system (13).

Macrophage function is strictly regulated by metabolites and metabolic pathways (70). For instance, when macrophages are activated in response to inflammatory signals, glycolytic metabolism is enhanced while Mitochondrial oxidative phosphorylation is attenuated (71, 72). The purpose of this metabolic reprogramming of inflammatory macrophages is to promote the rapid production of ATP, obtaining the energy and biosynthetic precursors necessary to fulfill their roles in the immune response, including phagocytosis and the production of inflammatory mediators (70).

### 3.2 Macrophages’ control over metabolism in adipose tissue

Different metabolic intermediates will drive adipose tissue macrophages, exhibiting polarization bias. In hypoxic environments, a significant amount of lactic acid will be generated during glycolysis, promoting macrophage replacement polarization (73–75). Adipose tissue macrophages that experience disruptions in lipid metabolism generate lipid droplets by encouraging the production of lysosomes, which exacerbates the inflammation inside the adipose tissue (76, 77). Adipokines are disrupted in an obese body, as demonstrated by increased expression of leptin and regulatory proteins, decreased secretion of lipocalin, which controls chemokine expression, and mast cell signaling, which controls glucose metabolism, polarization of macrophages to the M1 type, and pro-inflammatory effects (78, 79). On the other hand, adiponectin reduces inflammation by boosting M2-type macrophage polarization and suppressing TNF-α and IL-6 production (80). In conclusion, energy metabolism and lipid metabolism will control adipose tissue macrophages, influencing their own polarization and ultimately controlling the onset and progression of inflammation.

Additionally, it has been discovered that mitochondria, the hub of cellular metabolism, control the activity of immune cells. Simultaneously, the effects of mitochondrial dynamics on immune cell function have been extensively studied (81). Lipopolysaccharide (LPS)-stimulated macrophages exhibit deficient glucose metabolism, which lowers intracellular ATP levels, activates signaling pathways linked to the energy sensor AMPK, and encourages autophagy (82). While studies have demonstrated that mitochondrial autophagy differs from general autophagy in its regulation of adipose tissue and whole-body energy metabolism, mitochondrial-nucleotide-binding oligomerization domain, leucine-rich repeat and pyrin domain- containing 3 is a distinct route that results in BAT dysfunction (83). To put it briefly, the inflammatory response is influenced by LPS-stimulated modifications in macrophage metabolism, which are linked to adipose tissue energy metabolism and mitochondrial autophagy.

Apart from mitochondria, there has been a recent focus on the role of macrophage polarization in maintaining metabolic homeostasis, which is governed by calcium homeostasis. Adipose tissue-accumulating M1 macrophages are a primary source of proinflammatory cytokines that obstruct insulin signaling. Conversely, anti-inflammatory cytokines that increase insulin sensitivity are produced by M2 macrophages (84). Inflammation and compromised mitochondrial function result from dysregulation of mitochondrial Ca^2+^ homeostasis. Through its interaction with the MCU, Connexin 43 reduces the amount of succinate dehydrogenase (SDH) oxidation caused by Palmitic acid. Consequently, pro-inflammatory M1 macrophages become more polarized (85).

Nrf2 increases glucose absorption and mTOR signaling in M1 macrophages by suppressing IL-10 gene expression and secretion (86). Intrarenal adipose tissue mass is markedly influenced by macrophage-derived insulin-like growth factor-1 (IGF1) during thermogenic stress, and adipocyte differentiation and lipid storage capacity are tissue-specifically regulated by IGF1 *in vitro* (87). Furthermore, Glucose transporter type 1 (GLUT1) translocation and glycolytic enzyme gene expression are regulated by IL-10, which suppresses glycolytic flux (88).The principal ligand for the mineralocorticoid receptor (MR) is aldosterone. Improper activation of the MR can cause abnormal adipokine secretion, increased expression of inflammatory factors like TNF-α and IL-6, decreased adiponectin, recruitment of macrophages, and up-regulation of leptin (89, 90). Heme lysine demethylase 6a (Kdm6a)-specific knockout of histone lysine demethylase 2a (Kdm2a) mice leads to a considerable increase in energy expenditure, BAT activity, and macrophage M2 polarization (91, 92). Overall, pro-inflammatory macrophage polarization is controlled by macrophage metabolic reprogramming, which is linked to inflammatory factor expression, mitochondrial calcium homeostasis, and the regulation of adipocyte malfunction and obesity.

## 4 Macrophages regulate adipocyte energy metabolism

Chronic low-level innate immune system activation, which arises from an imbalance between energy intake and consumption, increased macrophage accumulation in adipose tissue, and dysfunction of adipose tissue, including decreased lipid storage capacity and lipogenesis, adipocyte necrosis, inflammation, insulin resistance, and fibrosis, are the hallmarks of obesity (93, 94). This demonstrates the fundamental role of macrophage-induced metabolic inflammation and the interaction between adipocytes and macrophages in obesity, and that understanding the pathophysiology of obesity is crucial for managing and preventing chronic metabolic disorders associated with obesity.

In order to eventually induce obesity-related metabolic diseases, hypertrophic adipocytes generate a lot of chemokines, attract immune cells, particularly macrophages, and create chronic low-grade inflammation and insulin resistance. They also release a lot of free fatty acids into the bloodstream (95).

Research indicates that the regulation of adipose tissue function in obesity is linked to innate immune cells (96). The earliest and most significant immune cells to be found in the adipose tissue of obese people are macrophages (97, 98). Macrophage infiltration is a primary cause of metabolic diseases associated with obesity and has a significant effect on the function of adipose tissue. Therefore, the prevention and treatment of obesity and other related metabolic illnesses depend on an understanding of the molecular mechanisms governing macrophage-adipocyte interactions in adipose tissue.

### 4.1 Macrophages regulate thermogenesis by modulating sympathetic neurochemicals

Thermogenesis is a mechanism used by all thermostatic organisms to keep their body temperature stable so that physiological functions and regular cell function can persist in cold environments. According to well-known thermogenic models, norepinephrine is released in brown and white adipose tissue when the hypothalamus detects hypothermia. This norepinephrine acts through β3-adrenergic receptors, promoting the expression of thermogenic genes and causing lipolysis in white adipocytes (99, 100).

#### 4.1.1 Macrophage modulates adipose tissue thermogenesis through influencing norepinephrine secretion

Reduced sympathetic innervation and local titers of norepinephrine are linked to BAT dysfunction in obese mice with macrophage mutations, which leads to a decrease in the production of thermogenic factors in adipocytes. Norepinephrine secretion and energy balance are regulated by signaling molecules such as Plexin4, TyrH, GCN2, and Slit3, which are expressed in macrophages (**Table 1**). The overexpression of PlexinA4 and the signaling receptor in the BAT-resident subset of Cx3Cr1 macrophages has been observed to result in the phenotypic rejection of sympathetic axons that express the transmembrane signaling protein Sema6A, hence hindering homeostatic thermogenesis (101).

**Table 1:** Signaling molecules that regulate NE release and affect homeostasis.

Association with macrophages	designation	Effects on energy homeostasis	References
Expressed in macrophages	Plexin4	Repels sympathetic node axons expressing the transmembrane protein Sema6A, reducing thermogenesis	(96)
	TyrH	CaMKIIγ upregulates TyrH basal phosphorylation to promote cold-induced norepinephrine production and increase thermogenesis	(97)
	GCN2	Increases the secretion of NE by macrophages, promotes browning of WAT and lipolysis	(98)
Secreted by M2-like macrophages	Slit3	Stimulates CaMKII signaling and NE release to increase thermogenesis	(99)
Promoting M2-like macrophage polarization	IL-4	Promotes the secretion of norepinephrine, increases thermogenesis	(100, 101)

**Figure 3:**
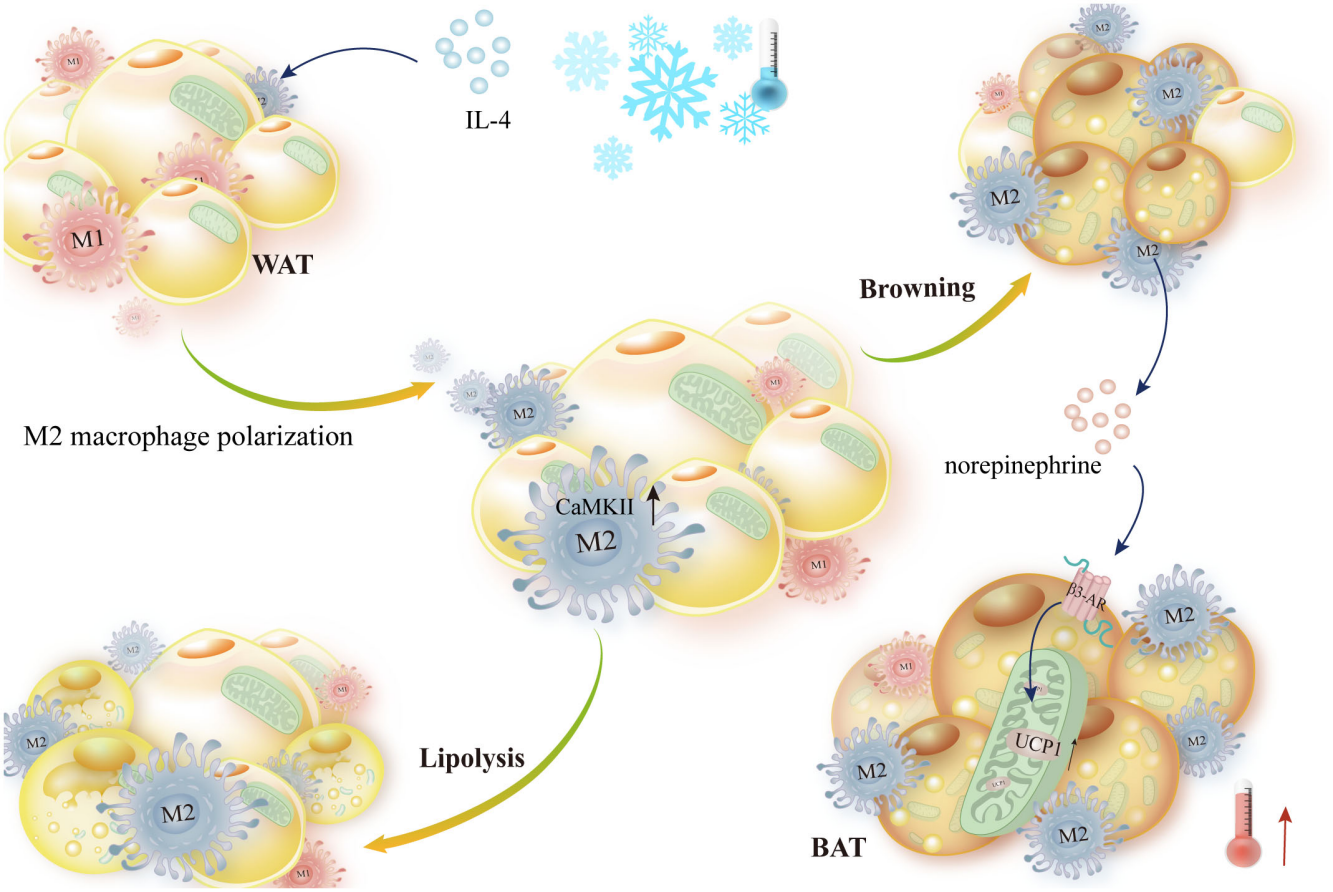
IL-4 stimulates norepinephrine-mediated thermogenesis and encourages macrophage alternative activation. IL-4 stimulated M2-like polarization of macrophages in WAT and BAT in response to cold stimulation. Then, norepinephrine secreted by M2-like macrophages activated β-AR-mediated lipolysis, upregulated UCP1 expression, and promoted thermogenesis, which was aided by elevated Ca2+/calmodulin-dependent protein kinase II expression.

Low temperatures quickly promote alternative macrophage activation in mouse adipose tissue. Following this, catecholamines are released by macrophages, which cause lipolysis in white adipose tissue and the expression of thermogenic genes in brown adipose tissue. Tyrosine hydroxylase is the enzyme that limits the pace at which catecholamines are produced, according to reports. Overexpression of constitutively active Ca^2+^/calmodulin-dependent protein kinase II (CaMKII) in macrophages increases *in vivo* adipose UCP1 expression and basal phosphorylation of TyrH. Additionally, CaMKII signaling enhances the production of catecholamines mediated by the cytokines IL-4 and IL-13 (102). In the inguinal WAT (iWAT) of mice exposed to cold, M2-like macrophages secrete a cytokine called Slit3, which binds to ROBO1 receptors on sympathetic neurons. This stimulation of CaMKII signaling and norepinephrine release increases adipocyte thermogenesis (103) (**Figure 3**).

It’s fascinating to note that bone marrow-derived macrophages do not release norepinephrine (NE) in response to IL-4 stimulation and that the conditioned media of IL-4-stimulated macrophages does not trigger expression of the thermogenic gene UCP1 in adipocytes cultivated with IL-4 (104). It’s debatable whether or not alternatively activated macrophages generate catecholamines and encourage adaptive thermogenesis in adipose tissue; this may have to do with the tissue selectivity of macrophages.

General control non-norepinephrine 2 kinase (GCN2) activation reduces the expression of monoamine oxidase A (MAOA), resulting in increased NE secretion by macrophages into adipocytes, subsequently enhancing WAT browning and lipolysis (105). Brown adipocytes exposed to norepinephrine, can express an inducible form of NOS similar to the iNOS from macrophage, and the induced NOS can promote NO generation and eventually improve vasodilation of the BAT microcirculation as well as thermogenesis (106).

It has been demonstrated that neuron-associated macrophages (SAM) also prevent adipose tissue browning and thermogenesis, in addition to adipose tissue macrophages. The deletion of the NE transporter solute carrier family 6 member 2 (SLC6A2) in mice results in an increase in brown adipose tissue (BAT), which browns white fat and increases thermogenesis. These two effects mitigate the symptoms associated with obesity in mice (107). In summary, reduced MAOA and SLC6A2 expression in macrophages stimulates NE production, which raises the browning and thermogenesis of adipose tissue.

Commonly expressed in adipose tissue among other tissues, adrenalomedullin 2 (ADM2) stimulates thermogenesis by raising AMPK and peroxisome proliferator-activated receptor gamma coactivator 1α (PGC1α) phosphorylation. This, in turn, stimulates the expression of UCP1 and ultimately thermogenesis in brown adipocytes (108). Furthermore, by inducing alternative M2 polarization in macrophages and activating the calcitonin receptor-like receptor (CRLR) RAMP1-cAMP/PKA and p38 MAPK pathways in white adipocytes, ADM2 directly stimulates subcutaneous fat metabolism (109). The alternative activation of macrophages attracted to cold-stressed subcutaneous white adipose tissue (scWAT) results in the induction of catecholamine production and tyrosine hydroxylase expression, both of which are necessary for scWAT browning (110). In summary, the AMPK, p38 MAPK signaling pathway, a crucial factor in WAT metabolism or browning, regulates macrophage alternative activation. This, in turn, stimulates adipose tissue thermogenesis and energy expenditure.

#### 4.1.2 Secretion of acetylcholine to promote thermogenesis

The primary players involved in controlling the activation of subcutaneous adipose thermogenesis are macrophages. Following an acute cold exposure, the number of cholinergic adipose macrophages (ChAM) was markedly elevated, and the cold-induced thermogenesis in mice was hindered by the deletion of choline acetyltransferase (ChAT), an enzyme involved in acetylcholine biosynthesis, from macrophages. Targeting metabolic disorders may benefit from the utilization of macrophages, which are a significant source of acetylcholine that controls adaptive thermogenesis in adipose tissue (111, 112). Overexpression of miR-182-5p in adipose tissue stimulates protein kinase A signaling, which in turn promotes the expression of thermogenic genes, enhances energy homeostasis, and increases adiposity, thermogenesis, and beige. It also induces FGF5 expression and acetylcholine secretion in adipocytes and activates nicotinic acetylcholine receptors in adipocytes (108). Overall, acetylcholine is a crucial mediator for macrophages to control adipose tissue thermogenesis, in addition to norepinephrine.

### 4.2 Macrophages regulate lipolysis and thermogenesis through inflammatory factors

Inflammatory factors and their mediators are thought to play a major part in the progression of metabolic disorders. For example, IL-25, IL-4, IL-13, IL-17, IL-10, and IL-1β are known to regulate the lipid deposition or breakdown of adipocytes, the browning of WAT, and the heat production of BAT, among other indirect regulatory effects (**Table 2**).

**Table 2:** Immune factors that regulate fat energy expenditure.

Association with macrophages	Inflammatory factors	Effects on energy homeostasis	References
Promoting M2-like macrophage polarization	IL-25	Promotes mitochondrial respiration and lipolysis	(109).
	IL-4, IL-13	Promotes WAT beiging	(107)
Derived from macrophages	NLRP3	Reduces UCP1 expression and inhibits thermogenesis	(110)
	IL-12	Inhibits BAT thermogenesis	(111)
	IL-1β	Induces mitochondrial dysfunction, inhibits the beiging of WAT	(110)

**Figure 4:**
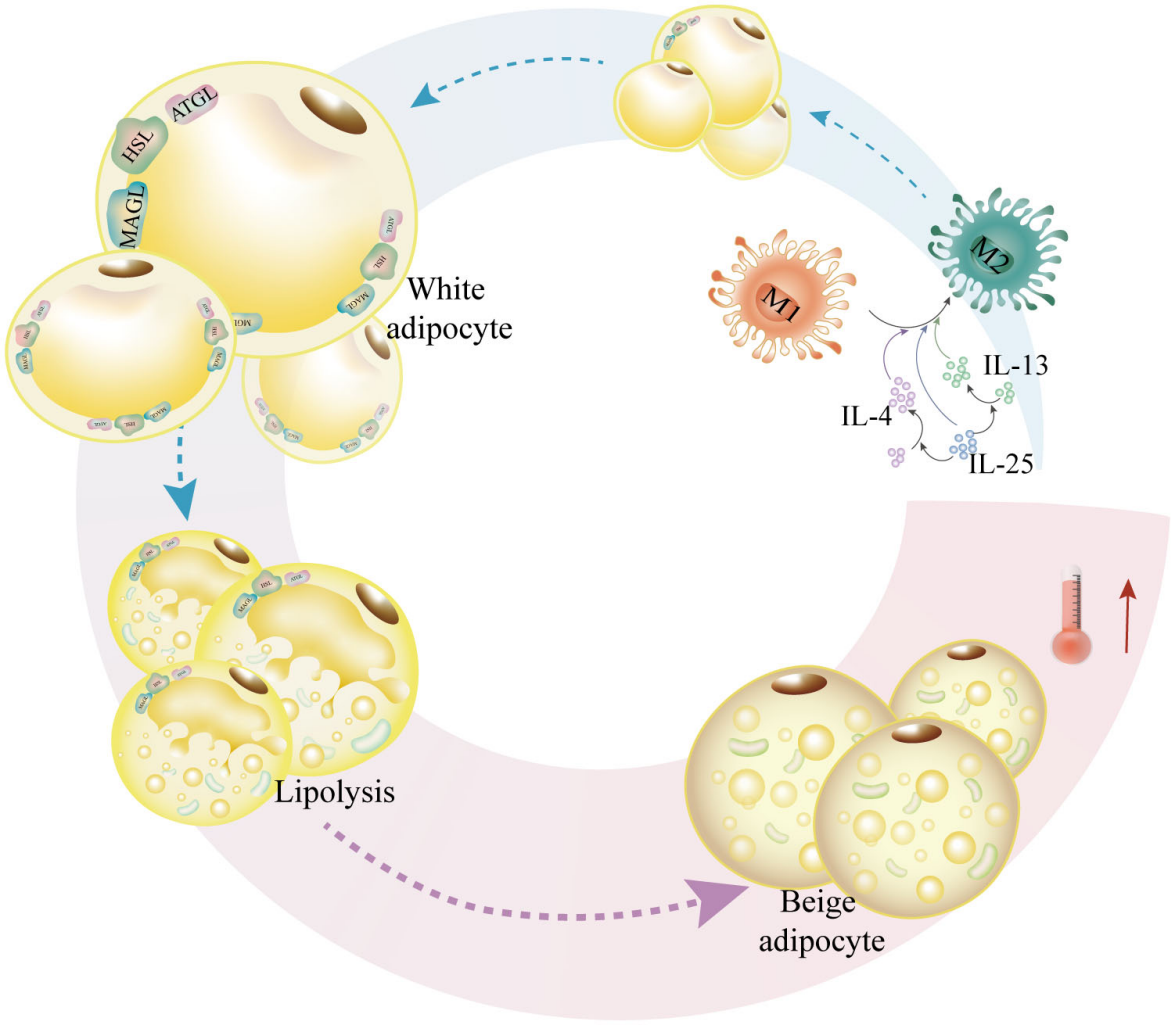
IL-4, IL-13, and IL-25 increase adipocytosis and militarization while also promoting M2-like macrophage polarization. In order to facilitate lipolysis, IL-25 causes macrophage M2-like polarization and upregulates the expression of lipases such as HSL, ATGL, and MAGL. In order to encourage adipocytosis, IL-25 also upregulates the expression of lipases, such as HSL, ATGL, and MAGL. Furthermore, IL-25 promotes M2-like macrophage-mediated adipocyte beiging and upregulates the production of IL-4 and IL-13.

IL-25 specifically induces M2 macrophage polarization, which enhances mitochondrial respiration and macrophage oxygen consumption rate by lowering fatty acid synthase, encouraging the expression of ATGL and HSL, and facilitating the expression of lipolytic, monoacylglycerol lipase (MAGL), and other adiponectin and β-oxidative enzymes. This results in increased NAD/NADH and ATP production, which in turn decreases weight gain and lipid accumulation (113). Furthermore, by releasing IL-4 and IL-13 and encouraging alternate macrophage activation, IL-25 causes beige adipose tissue accumulation in WAT (111). In perspective, IL-4, IL-13, and IL-25 can simply lower the expression of adipose synthase, increase lipolysis and adipocyte growth, and promote macrophage M2 polarization (**Figure 4**).

Moreover, by blocking the lipogenic gene PPARγ, the IL-17 subfamily of IL-25 inhibits adipogenesis and controls adipose differentiation. Conversely, elevated ILC2 or IL-33 translocation stimulates browning *in vivo*, while ILC2 upregulates UCP1 expression *in vitro* (114). It is unclear, therefore, if IL-17 has any effect on the control of adipogenesis and differentiation, which has an additional impact on thermogenesis and fat browning. Vascular endothelial growth factor indirectly affects M2 macrophage polarization, and the browning of WAT controls thermogenic activity, which raises energy expenditure, improves insulin sensitivity, and ultimately enhances systemic metabolism (115–117). To sum up, cold stimulation increases the expression of adipose-vascular endothelial growth factor, which controls M2 macrophage polarization and hence enhances browning of the adipose tissue.

Unexpectedly, more research has revealed that total macrophages (MΦs) stimulated by IL10/TGFβ express low amounts of CD40 and high levels of CD163, and they also release chemicals that stop white adipocytes’ ATP-related respiration. Adipocytes with low CD163 expression and high CD40 expression had increased mitochondrial activity due to chemicals generated by LPS/IFNγ-activated MΦs (118). This suggests that whereas M2 macrophages block mitochondrial respiration, M1 macrophages behave in a counteractive manner. In conclusion, it appears that resident macrophages control energy metabolism in human adipocytes through an activation-dependent paracrine mechanism. Nevertheless, the precise variations in resident macrophage regulation of energy metabolism in various tissues require additional research.

NLRP3 activation in macrophages decreases mitochondrial respiration and UCP1 induction in primary adipocyte cultures (119). Other research indicates that fat-sensing macrophages send this signal to obese hepatocytes, which then release FGF21 to promote BAT thermogenesis. On the other hand, in the absence of p38 activation, there is an increase in IL-12 release in macrophages, which suppresses FGF21 and lowers BAT thermogenesis (120). This suggests that proinflammatory factors and components associated with macrophages will be expressed more frequently, and then adipocyte browning and thermogenesis will be suppressed.

### 4.3 Macrophage crosstalk affects mitochondrial function to regulate energy homeostasis

In response to external stimuli, macrophages polarize into distinct metabolic forms that alter, generating various intermediate products, impacting mitochondrial biogenesis, mitochondrial transfer, browning of white fat, and ultimately thermogenesis and energy homeostasis.

**Figure 5:**
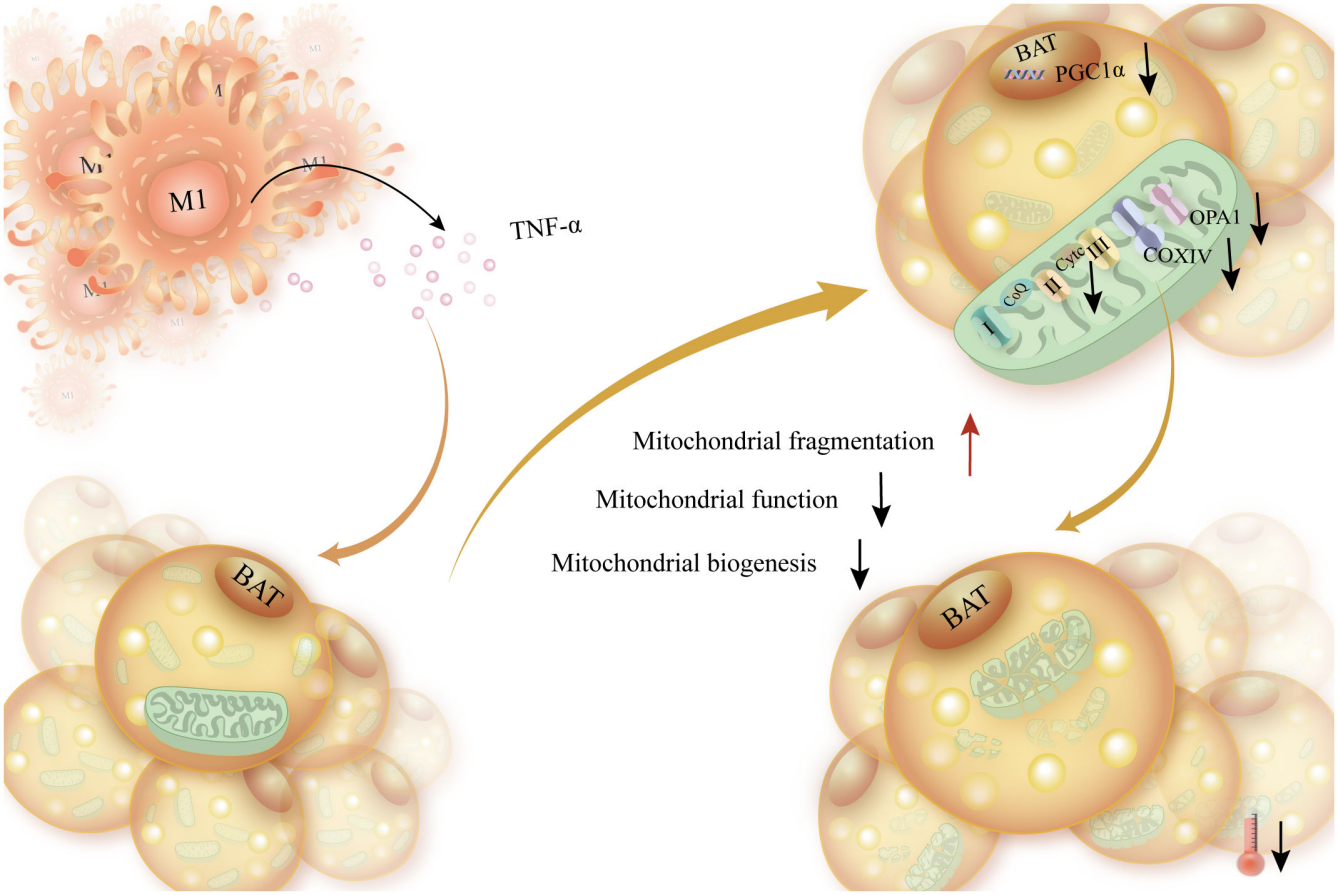
The elevated production of TNF-a released by macrophages disrupts energy expenditure, enhances mitochondrial fragmentation in brown adipocytes, and affects mitochondrial function. Increased TNF-α secreted by macrophages caused brown adipocytes to produce less ATP and less PGC-1α, the transcription factor that synthesizes mitochondrial biosynthesis. This, in turn, caused brown adipocytes to produce less mitochondrial biosynthesis, impaired mitochondrial function, and increased mitochondrial disruption.

Mitochondrial failure has been reported to be associated with a decrease in the insulin receptor substrate 1 (IRS1) gene due to a decrease in the number of M2 macrophages and an increase in adipogenic genes such as acetyl coenzyme A carboxylase α (ACACA), fatty acid synthase (FASN), and thyroid hormone responsiveness (THRSP) (121). Adipocytes’ mitochondrial bioenergetics were markedly changed following exposure to TNF-α, exhibiting increased proton leakage, increased basal respiration, and decreased ATP conversion. Furthermore, a decline in cytochrome c oxidase IV (COX IV) and cytochrome complex (CytC) expression coincided with the reduction in peroxisome prolilerators-activated receptor γ coactivator lalpha (PGC-1α) mRNA expression. Reduced mitochondrial activity and adipocyte reprogramming were the outcomes of TNF-α (122). Additionally, it was discovered that deleting TLR4 specifically in hematopoietic cells improved homeostasis in perivascular adipose tissue, which decreased TNF-α release triggered by macrophages and increased mitochondrial biogenesis in brown adipocytes (123). To sum up, elevated production of TNF-α by macrophages suppresses thermogenesis via impeding mitochondrial biogenesis (**Figure 5**).

**Figure 6:**
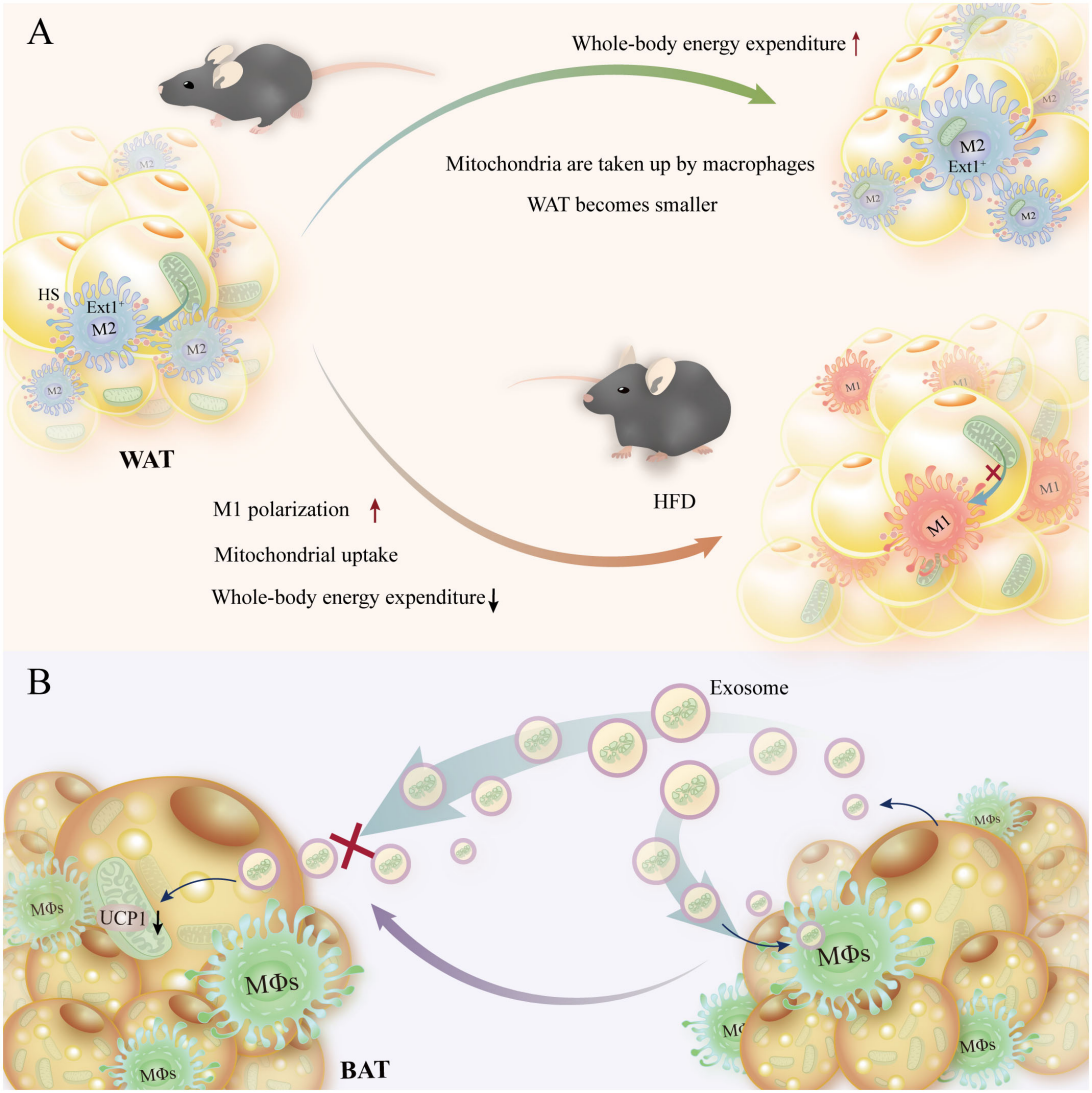
Energy homeostasis is preserved, and homeostatic energy expenditure is increased when macrophages receive mitochondrial transfer from adipocytes. **(A)** Shrinkage of adipocytes and increased organismal energy expenditure are facilitated by high HS-expressing Ext1+ macrophages in adipocytes, which promote mitochondrial translocation to macrophages. In contrast, reduced HS expression with M1-type macrophages slows mitochondrial translocation and reduces organismal energy expenditure in mice on a high-fat diet. **(B)** Macrophage phagocytosis of exosomes containing mitochondrial debris prevents reabsorption of brown adipocytes, which in turn decreases mitochondrial UCP levels.

It was shown that mitochondrial absorption requires the heparan sulfate (HS) biosynthesis pathway, which is crucial in preserving lipid and glucose homeostasis in humans and animals. Ext1 (the gene responsible for HS biosynthesis) deletion causes an increase in WAT mass, a decrease in energy expenditure, a reduction in the transfer of mitochondria from adipocytes to macrophages within cells, and an aggravation of *in vivo* obesity brought on by a high-fat diet (124). In addition, macrophage polarization has a major impact on macrophage migration in adipose tissue. M1-type macrophage polarization in obese patients limits mitochondrial uptake, whereas M2-type macrophage polarization promotes mitochondrial transfer to macrophages in adipose tissue (33, 125). The information above also implies that high expression of HS and macrophage alternative activation enhance macrophage mitochondrial uptake and increase energy expenditure. In addition to directly phagocytosing extracellular matrix (EVs) containing mitochondrial pieces, macrophages also phagocytose EVs to prevent the thermogenic effects of BAT. Brown adipocyte macrophages extract EVs that contain pieces of mitochondria. By preventing the drop in mitochondrial protein UCP1 and peroxisome proliferator-activated receptor γ signaling caused by brown adipocytes reabsorbing EVs, this prevents the thermogenesis process from failing (126) (**Figure 6**).

This suggests that adipocytes and macrophages depend on intercellular mitochondrial transfer as a mechanism of immune metabolic crosstalk to regulate metabolic disorders in obesity, but the mechanism of mitochondrial transfer is unclear and needs to be further studied. In addition, macrophages participate in the mitochondrial quality control (MQC) system by removing extracellular mitochondrial debris to maintain the thermogenic function of BAT (127).

Moreover, in order to preserve BAT’s thermogenic activity, macrophages remove extracellular mitochondrial debris as part of the MQC system (127). The mechanism of intercellular mitochondrial transfer is unclear and requires more research; however, these data indicate that there is an immune metabolic crosstalk between adipocytes and macrophages to regulate metabolic abnormalities in obesity.

### 4.4 Macrophage regulates fatty acid oxidation

Ablation of adipocyte fatty acid-binding protein (FABP4/aP2) in macrophages leads to increased expression of uncoupling proteins 2 and Sirt3, increased oxygen production, lipopolysaccharide-induced mitochondrial dysfunction, and fatty acid oxidation, leading to an anti-inflammatory state *in situ* and *in vivo* (128). FABP4/aP2 regulates macrophage redox signaling and inflammosome activation by controlling UCP2 expression (129). Peanut sprouts (PS) inhibit triglyceride accumulation in adipocytes through fatty acid oxidation, whereas PSE extracts (PSEs) in macrophages and adipocytes impede LPS-mediated inflammation induction. PSE also prevents LPS-induced inhibition of adipocyte browning and activates mitochondria in Bt-cAMP-treated adipocytes (130).

Co-culture of adipocytes and macrophages stimulates endogenous fatty acid released from adipocytes via β3 adrenergic stimulation, leading to activation of NF-κB, a major regulator of the inflammatory response in both cell types. Pharmacological inhibition of NF-κB significantly inhibits co-culture-induced production of pro-inflammatory factors and adipocyte lipolysis (131). Short-chain fatty acids (SCFA) activate free fatty acid receptor 2 (Ffar2), also called G protein-coupled receptor (GPR43), which is found in the gut, adipocytes, and immune cells. It is involved in the regulation of lipids and the immune system. Knockout of Ffar2 in the HFD-treated mice can enhance thermogenesis and energy expenditure and reduce macrophage concentration in the WAT (132). In summary, elevated fatty acid oxidation in macrophages prevents adipocyte thermogenesis and causes mitochondrial dysfunction.

### 4.5 Macrophage regulates glycolysis to influence thermogenesis

Since M1 macrophages rely heavily on fatty acid oxidation, glycolysis is the main source of energy for them (133, 134). PPARγ and STAT6 have been demonstrated to be regulated by pyruvate kinase isoform M2 (PKM2), which also stimulates M2-type differentiation and macrophage activation. These events ultimately trigger a metabolic transition from glycolysis to fatty acid oxidation (135–137). When the macrophages are metabolically activated, they will exhibit higher oxidative phosphorylation (OXPHOS) and glycolysis. Co-culturing macrophages with adipocytes also induces increased cytokine production in macrophages and overexpression of glycolysis-related proteins (138). Adipocyte-produced soluble factor adiponectin inhibits naïve T cells from developing into Th1 and Th17 cells and reduces CD4 T cells positive for IL-17 and IFN-γ in HFD-treated mice. Adiponectin suppresses glycolysis and reduces Th17 cell development in an adenosine 5’-monophosphate-activated protein kinase-dependent manner (139). Triggering receptor expressed on myeloid cells 2 (Trem2) expression in macrophages prevents the progression of metabolic disorders by promoting the formation of coronary structures around lipid-rich and cell-dead adipocytes, restricting fat cell hypertrophy, and decreasing cholesterol levels in mice fed a high-fat diet (140).

**Figure 7:**
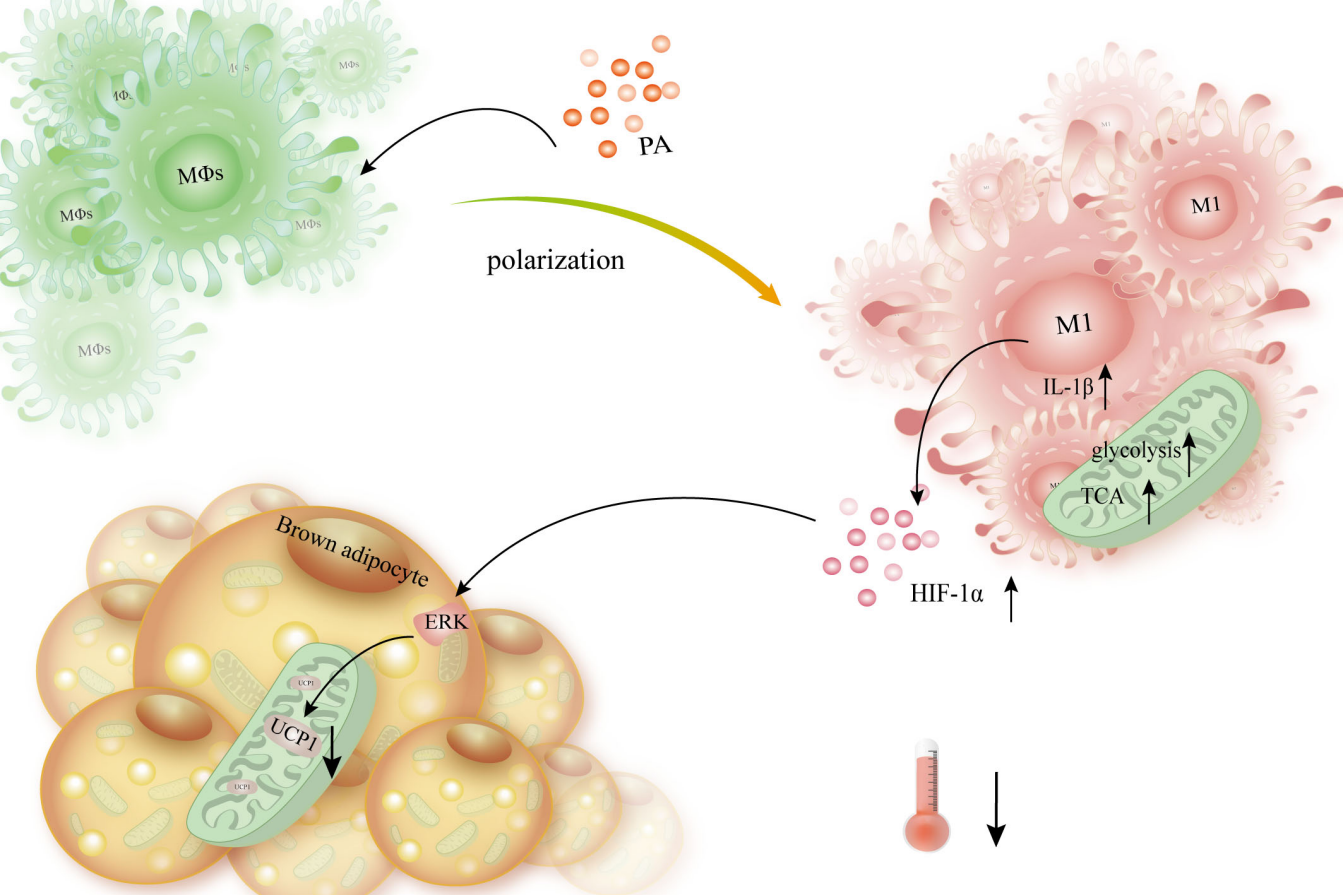
Macrophages release HIF-1α and IL-1β, which slows thermogenesis and lowers the expression of UCP1 in brown adipocytes. When PA interacts with macrophages, it causes them to polarize into the M1 type, which is followed by increased HIF-1α expression, improved TCA, and enhanced glycolysis. Simultaneously, it boosted IL-1β expression, triggered ERK, and subsequently suppressed β-AR-mediated UCP1 production, hence diminishing BAT’s thermogenesis and energy expenditure.

Following lipolysis in obese mice, macrophages profoundly infiltrate adipocytes (97). In palmitate-treated macrophages, hypoxia stimulates JNK and p38 MAPK expression (141). Palmitate not only facilitates macrophages to express more HIF-1α but also promotes the polarization of macrophages to M1-type macrophages, as well as the increased level of IL-1β and genes involved in the palmitate-induced glycolysis pathway. This enhancement of glycolysis causes an increase in lactic acid secretion and succinate, a TCA product (142, 143). The buildup of succinate results in an inflammatory signal through HIF-1α that increases the production of IL-1β (144). The upregulated mRNA level of IL-1β in WAT and macrophages from obese mice can inhibit the activation of extracellular signal-regulated kinase (ERK) in adipocytes and block the UCP1 production stimulated by the β-adrenergic receptor (145). In summary, palmitate promotes macrophage M1 polarization, expresses more HIF-1α as well as IL-1β, and promotes glycolysis (**Figure 7**).

## 5 Adipocytes regulate energy homeostasis by adipokines crosstalk macrophages

In addition to lipid molecules, adipocytes release peptides known as adipokines, such as leptin, adiponectin, retinol binding protein4 (RBP4), fatty acid binding protein 4 (FABP4), and tumor necrosis factor. These elements can regulate metabolism by acting either locally (paracrine) on nearby cells or remotely (endocrine) on cells in different organs (37). Colony-stimulating factor 1 receptor (CSF1R) inhibition and germline deletion by altering macrophage function, it has been reported that Trib1, Slc6a2, Csf1r, Ccr2, or Trem2 may play direct or indirect roles in the control of energy storage (140, 146–148). The cytokine globular adiponectin, which originates from adipocytes, induces macrophage mitochondria to produce reactive oxygen species, which in turn sets off an apoptotic cascade (149).

**Figure 8:**
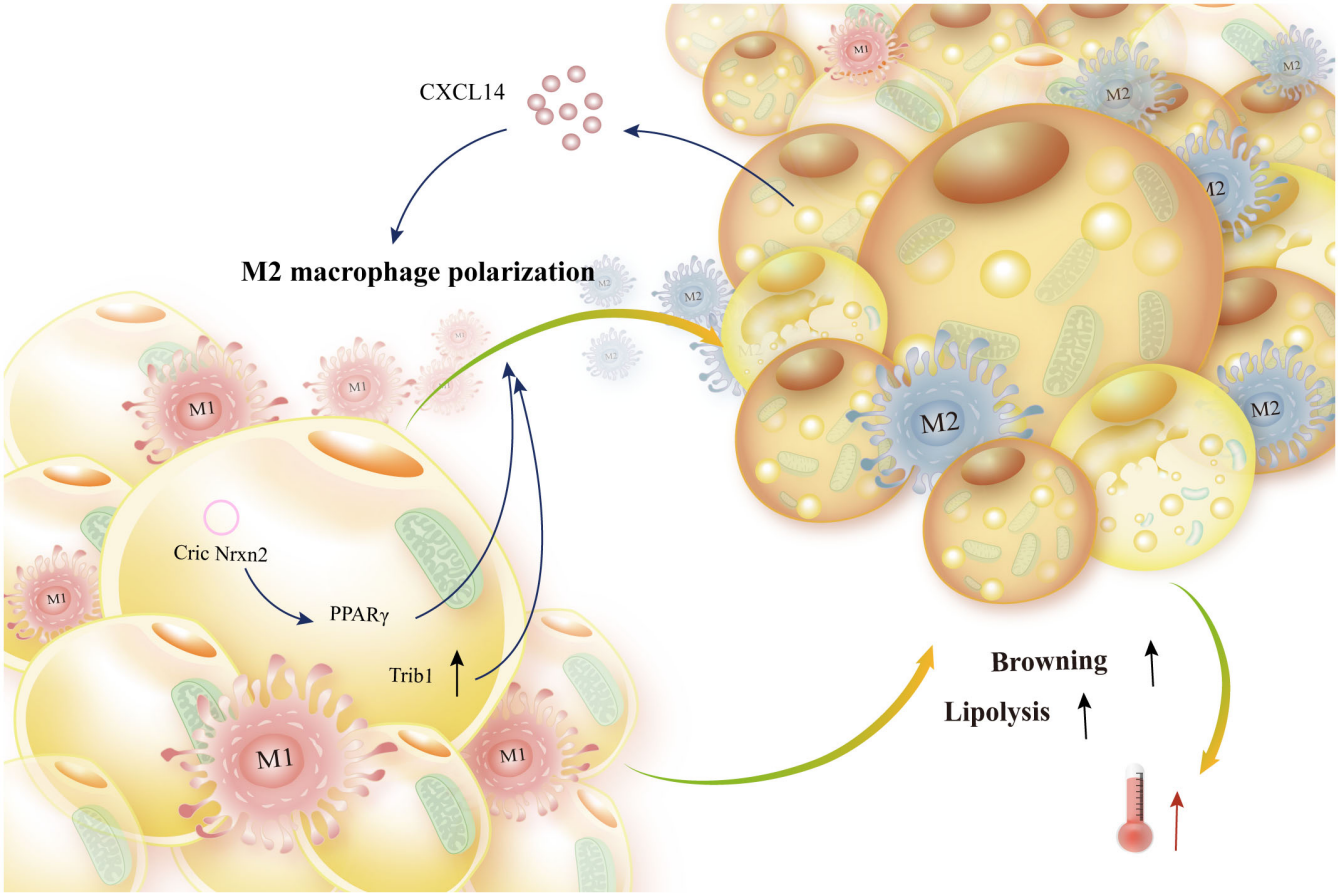
Adipokine production from adipocytes encourages lipolysis and browning. Brown adipocyte-secreted CXCL14 and white adipocyte-produced CircNrxn2 mediate PPARγ to induce M2-like macrophage polarization. Additionally, trib1 promotes alternative macrophage activation, which enhances energy expenditure and browning and lipolysis of adipocytes.

In addition, by promoting lipolysis, easing insulin resistance and hypertriglyceridemia, promoting M2-type differentiation of adipose tissue-resident macrophages, and lowering the expression of pro-inflammatory markers, Trib1 preserves metabolic homeostasis (146). The study found that circNrxn2 enhances M2-type macrophage polarization, inhibits the activity of miR-103, and lessens its regulation of fibroblast growth factor 10 (FGF10) production by regulating the PPARγ signaling pathway and improving adipose browning (150). Brown adipokine growth and differentiation factor 15 (GDF15) targets macrophages and suppresses the production of pro-inflammatory genes, hence enhancing thermogenesis during thermal activity (151) (**Figure 8**). Adiponectin, a plentiful adipogen, binds to T-adhesins on the surface of M2 macrophages to attract them. Adiponectin stimulates beige cells and promotes cell proliferation by activating Akt (152). Brown adipocytes release CXCL14, which draws surrogate-activated (M2) macrophages. These macrophages then activate BAT and white adipocytes, hence advancing adaptive thermogenesis (153).

## 6 Conclusion

Recent studies have shown that immunocytes play an important role in metabolic homeostasis, and the accompanying immune metabolism is becoming a focus of interest. Macrophages, with their enormous plasticity, functional diversity, and tissue specificity in immunity and energy homeostasis, have become another interesting object of study within the immune cell population. Several studies have shown that lipid metabolism affects macrophage activity; however, macrophages inversely regulate lipid metabolism. Therefore, it is necessary to study how adipocytes and macrophages interact to maintain energy homeostasis in metabolic diseases such as obesity.

**Figure 9:**
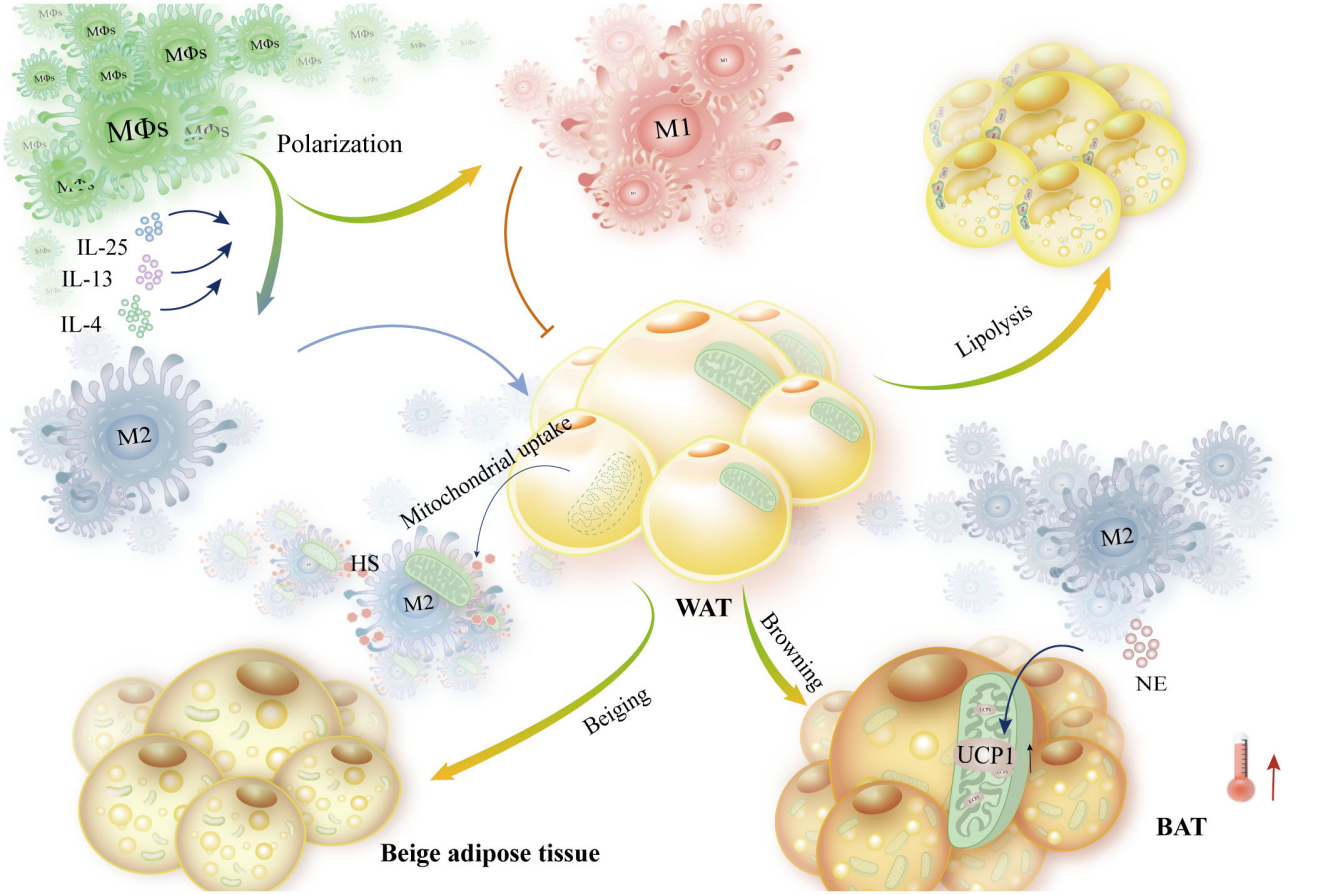
Adipose tissue metabolism is impacted by macrophage polarization bias, which also controls energy homeostasis. Macrophages exposed to IL-4, IL-13, and IL-15 polarized to the M2 type. Furthermore, NE is secreted by M2-like macrophages to enhance browning and elevate UCP1 expression, which in turn increases thermogenesis. These macrophages also support lipolysis and WAT metabolism. High-HS-expressing macrophages increase energy expenditure and mitochondrial translocation of WAT. M1-like macrophages, on the other hand, prevent these outcomes.

According to this article, macrophages influence the β-AR-mediated thermogenesis of fat cells, control the release of norepinephrine and acetylcholine, and collaborate with the central nervous system. Inflammatory factors such as IL-4, IL-13, IL-1β, and IL-25 indirectly interfere with macrophages, which in turn affects fat remodeling and thermogenesis. The majority of this interference is accomplished by altering macrophage polarization. Some research indicates that M2-like macrophage polarization enhances energy expenditure. Thus, controlling macrophage M2-like polarization is undoubtedly helpful in preserving energy homeostasis in metabolic disorders like obesity (**Figure 9**). Reversely, by secreting adipokines such as Trib1, GDF15, and CXCL14, adipocytes can suppress the expression of macrophage inflammatory factors. Additionally, they can stimulate M2-like macrophage polarization, which intensifies adipocyte browning and heat production. At the same time, macrophage polarization alters metabolic rhythms, affects thermogenesis mediated by the central nervous system, and indirectly controls lipolysis and adipocyte browning. Through physical phagocytosis, macrophages also influence adipocyte uptake of mitochondria, thereby altering the regulation of cellular thermogenesis and ultimately reducing the risk of metabolic diseases such as obesity. The complexity and variability of the immune microenvironment has led to complications in studying the interactions between immune and parenchymal cells. However, macrophages are critical in metabolic diseases, and so this paper describes the molecular mechanisms of adipocyte-macrophage interactions, which informs the search for new strategies and therapeutic targets to maintain energy homeostasis.

## Author contributions

YZ: Writing – original draft. BZ: Writing – review & editing. XS: Writing – review & editing.

## References

[1] Swinburn BA Sacks G Hall KD McPherson K Finegood DT Moodie ML. The global obesity pandemic: shaped by global drivers and local environments. Lancet. (2011) 378:804–14. doi: 10.1016/S0140-6736(11)60813-1 21872749

[2] NCD Risk Factor Collaboration (NCD-RisC). Worldwide trends in body-mass index, underweight, overweight, and obesity from 1975 to 2016: a pooled analysis of 2416 population-based measurement studies in 128·9 million children, adolescents, and adults. Lancet. (2017) 390:2627–42. doi: 10.1016/S0140-6736(17)32129-3 PMC5735219 29029897

[3] Safaei M Sundararajan EA Driss M Boulila W Shapi’i A. A systematic literature review on obesity: Understanding the causes & consequences of obesity and reviewing various machine learning approaches used to predict obesity. Comput Biol Med. (2021) 136:104754. doi: 10.1016/j.compbiomed.2021.104754 34426171

[4] Cannon B Nedergaard J. Brown adipose tissue: function and physiological significance. Physiol Rev. (2004) 84:277–359. doi: 10.1152/physrev.00015.2003 14715917

[5] Zechner R Zimmermann R Eichmann TO Kohlwein SD Haemmerle G Lass A. FAT SIGNALS–lipases and lipolysis in lipid metabolism and signaling. Cell Metab. (2012) 15:279–91. doi: 10.1016/j.cmet.2011.12.018 PMC3314979 22405066

[6] Bartelt A Heeren J. Adipose tissue browning and metabolic health. Nat Rev Endocrinol. (2014) 10:24–36. doi: 10.1038/nrendo.2013.204 24146030

[7] Shao M Wang QA Song A Vishvanath L Busbuso NC Scherer PE. Cellular origins of beige fat cells revisited. Diabetes. (2019) 68:1874–85. doi: 10.2337/db19-0308 PMC6754244 31540940

[8] Li JH Hepworth MR O’Sullivan TE. Regulation of systemic metabolism by tissue-resident immune cell circuits. Immunity. (2023) 56:1168–86. doi: 10.1016/j.immuni.2023.05.001 PMC10321269 37315533

[9] Frayn KN Karpe F Fielding BA Macdonald IA Coppack SW. Integrative physiology of human adipose tissue. Int J Obes Relat Metab Disord. (2003) 27:875–88. doi: 10.1038/sj.ijo.0802326 12861227

[10] Nance SA Muir L Lumeng C. Adipose tissue macrophages: Regulators of adipose tissue immunometabolism during obesity. Mol Metab. (2022) 66:101642. doi: 10.1016/j.molmet.2022.101642 36402403 PMC9703629

[11] Gordon S Taylor PR. Monocyte and macrophage heterogeneity. Nat Rev Immunol. (2005) 5:953–64. doi: 10.1038/nri1733 16322748

[12] Sunderkötter C Nikolic T Dillon MJ Van Rooijen N Stehling M Drevets DA. Subpopulations of mouse blood monocytes differ in maturation stage and inflammatory response. J Immunol. (2004) 172:4410–7. doi: 10.4049/jimmunol.172.7.4410 15034056

[13] Mosser DM Edwards JP. Exploring the full spectrum of macrophage activation. Nat Rev Immunol. (2008) 8:958–69. doi: 10.1038/nri2448 PMC2724991 19029990

[14] Nahrendorf M Swirski FK Aikawa E Stangenberg L Wurdinger T Figueiredo J-L. The healing myocardium sequentially mobilizes two monocyte subsets with divergent and complementary functions. J Exp Med. (2007) 204:3037–47. doi: 10.1084/jem.20070885 PMC2118517 18025128

[15] Takahashi K Mizuarai S Araki H Mashiko S Ishihara A Kanatani A. Adiposity elevates plasma MCP-1 levels leading to the increased CD11b-positive monocytes in mice. J Biol Chem. (2003) 278:46654–60. doi: 10.1074/jbc.M309895200 13129912

[16] Chen A Mumick S Zhang C Lamb J Dai H Weingarth D. Diet induction of monocyte chemoattractant protein-1 and its impact on obesity. Obes Res. (2005) 13:1311–20. doi: 10.1038/oby.2005.159 16129712

[17] Kanda H Tateya S Tamori Y Kotani K Hiasa K Kitazawa R. MCP-1 contributes to macrophage infiltration into adipose tissue, insulin resistance, and hepatic steatosis in obesity. J Clin Invest. (2006) 116:1494–505. doi: 10.1172/JCI26498 PMC1459069 16691291

[18] Lumeng CN Bodzin JL Saltiel AR. Obesity induces a phenotypic switch in adipose tissue macrophage polarization. J Clin Invest. (2007) 117:175–84. doi: 10.1172/JCI29881 PMC1716210 17200717

[19] Wynn TA Vannella KM. Macrophages in tissue repair, regeneration, and fibrosis. Immunity. (2016) 44:450–62. doi: 10.1016/j.immuni.2016.02.015 PMC4794754 26982353

[20] Lumeng CN DelProposto JB Westcott DJ Saltiel AR. Phenotypic switching of adipose tissue macrophages with obesity is generated by spatiotemporal differences in macrophage subtypes. Diabetes. (2008) 57:3239–46. doi: 10.2337/db08-0872 PMC2584129 18829989

[21] Lee YS Wollam J Olefsky JM. An integrated view of immunometabolism. Cell. (2018) 172:22–40. doi: 10.1016/j.cell.2017.12.025 29328913 PMC8451723

[22] Russo L Lumeng CN. Properties and functions of adipose tissue macrophages in obesity. Immunology. (2018) 155:407–17. doi: 10.1111/imm.13002 PMC6230999 30229891

[23] Viola A Munari F Sánchez-Rodríguez R Scolaro T Castegna A. The metabolic signature of macrophage responses. Front Immunol. (2019) 10:1462. doi: 10.3389/fimmu.2019.01462 31333642 PMC6618143

[24] Morris DL Singer K Lumeng CN. Adipose tissue macrophages: phenotypic plasticity and diversity in lean and obese states. Curr Opin Clin Nutr Metab Care. (2011) 14:341–6. doi: 10.1097/MCO.0b013e328347970b PMC4690541 21587064

[25] Ahmed B Sultana R Greene MW. Adipose tissue and insulin resistance in obese. BioMed Pharmacother. (2021) 137:111315. doi: 10.1016/j.biopha.2021.111315 33561645

[26] Zhang T Chen J Tang X Luo Q Xu D Yu B. Interaction between adipocytes and high-density lipoprotein:new insights into the mechanism of obesity-induced dyslipidemia and atherosclerosis. Lipids Health Dis. (2019) 18:223. doi: 10.1186/s12944-019-1170-9 31842884 PMC6913018

[27] Polyzos SA Kountouras J Mantzoros CS. Obesity and nonalcoholic fatty liver disease: From pathophysiology to therapeutics. Metabolism. (2019) 92:82–97. doi: 10.1016/j.metabol.2018.11.014 30502373

[28] Zatterale F Raciti GA Prevenzano I Leone A Campitelli M De Rosa V. Epigenetic reprogramming of the inflammatory response in obesity and type 2 diabetes. Biomolecules. (2022) 12:982. doi: 10.3390/biom12070982 35883538 PMC9313117

[29] Kershaw EE Flier JS. Adipose tissue as an endocrine organ. J Clin Endocrinol Metab. (2004) 89:2548–56. doi: 10.1210/jc.2004-0395 15181022

[30] Frühbeck G Gómez-Ambrosi J Muruzábal FJ Burrell MA. The adipocyte: a model for integration of endocrine and metabolic signaling in energy metabolism regulation. Am J Physiol Endocrinol Metab. (2001) 280:E827–847. doi: 10.1152/ajpendo.2001.280.6.E827 11350765

[31] Zhang Y Proenca R Maffei M Barone M Leopold L Friedman JM. Positional cloning of the mouse obese gene and its human homologue. Nature. (1994) 372:425–32. doi: 10.1038/372425a0 7984236

[32] Sanchez-Gurmaches J Guertin DA. Adipocyte lineages: tracing back the origins of fat. Biochim Biophys Acta. (2014) 1842:340–51. doi: 10.1016/j.bbadis.2013.05.027 PMC3805734 23747579

[33] Brestoff JR Artis D. Immune regulation of metabolic homeostasis in health and disease. Cell. (2015) 161:146–60. doi: 10.1016/j.cell.2015.02.022 PMC4400287 25815992

[34] Cohen P Kajimura S. The cellular and functional complexity of thermogenic fat. Nat Rev Mol Cell Biol. (2021) 22:393–409. doi: 10.1038/s41580-021-00350-0 33758402 PMC8159882

[35] Kajimura S Seale P Kubota K Lunsford E Frangioni JV Gygi SP. Initiation of myoblast to brown fat switch by a PRDM16-C/EBP-beta transcriptional complex. Nature. (2009) 460:1154–8. doi: 10.1038/nature08262 PMC2754867 19641492

[36] Kwok KHM Lam KSL Xu A. Heterogeneity of white adipose tissue: molecular basis and clinical implications. Exp Mol Med. (2016) 48:e215. doi: 10.1038/emm.2016.5 26964831 PMC4892883

[37] Morigny P Boucher J Arner P Langin D. Lipid and glucose metabolism in white adipocytes: pathways, dysfunction and therapeutics. Nat Rev Endocrinol. (2021) 17:276–95. doi: 10.1038/s41574-021-00471-8 33627836

[38] Sidossis L Kajimura S. Brown and beige fat in humans: thermogenic adipocytes that control energy and glucose homeostasis. J Clin Invest. (2015) 125:478–86. doi: 10.1172/JCI78362 PMC4319444 25642708

[39] Wu L Zhang L Li B Jiang H Duan Y Xie Z. AMP-activated protein kinase (AMPK) regulates energy metabolism through modulating thermogenesis in adipose tissue. Front Physiol. (2018) 9:122. doi: 10.3389/fphys.2018.00122 29515462 PMC5826329

[40] Herz CT Kiefer FW. Adipose tissue browning in mice and humans. J Endocrinol. (2019) 241:R97–R109. doi: 10.1530/JOE-18-0598 31144796

[41] Park J Shin S Liu L Jahan I Ong S-G Xu P. Progenitor-like characteristics in a subgroup of UCP1+ cells within white adipose tissue. Dev Cell. (2021) 56:985–999.e4. doi: 10.1016/j.devcel.2021.02.018 33711247 PMC8026751

[42] Wang QA Tao C Gupta RK Scherer PE. Tracking adipogenesis during white adipose tissue development, expansion and regeneration. Nat Med. (2013) 19:1338–44. doi: 10.1038/nm.3324 PMC4075943 23995282

[43] Chouchani ET Kazak L Spiegelman BM. New advances in adaptive thermogenesis: UCP1 and beyond. Cell Metab. (2019) 29:27–37. doi: 10.1016/j.cmet.2018.11.002 30503034

[44] Ikeda K Kang Q Yoneshiro T Camporez JP Maki H Homma M. UCP1-independent signaling involving SERCA2b-mediated calcium cycling regulates beige fat thermogenesis and systemic glucose homeostasis. Nat Med. (2017) 23:1454–65. doi: 10.1038/nm.4429 PMC5727902 29131158

[45] Cypess AM Lehman S Williams G Tal I Rodman D Goldfine AB. Identification and importance of brown adipose tissue in adult humans. N Engl J Med. (2009) 360:1509–17. doi: 10.1056/NEJMoa0810780 PMC2859951 19357406

[46] Senthivinayagam S Serbulea V Upchurch CM Polanowska-Grabowska R Mendu SK Sahu S. Adaptive thermogenesis in brown adipose tissue involves activation of pannexin-1 channels. Mol Metab. (2021) 44:101130. doi: 10.1016/j.molmet.2020.101130 33248294 PMC7779784

[47] Oeckl J Janovska P Adamcova K Bardova K Brunner S Dieckmann S. Loss of UCP1 function augments recruitment of futile lipid cycling for thermogenesis in murine brown fat. Mol Metab. (2022) 61:101499. doi: 10.1016/j.molmet.2022.101499 35470094 PMC9097615

[48] Boström P Wu J Jedrychowski MP Korde A Ye L Lo JC. A PGC1-α-dependent myokine that drives brown-fat-like development of white fat and thermogenesis. Nature. (2012) 481:463–8. doi: 10.1038/nature10777 PMC3522098 22237023

[49] Zhang Z Zhang H Li B Meng X Wang J Zhang Y. Berberine activates thermogenesis in white and brown adipose tissue. Nat Commun. (2014) 5:5493. doi: 10.1038/ncomms6493 25423280

[50] Xu Y Yu T Ma G Zheng L Jiang X Yang F. Berberine modulates deacetylation of PPARγ to promote adipose tissue remodeling and thermogenesis *via* AMPK/SIRT1 pathway. Int J Biol Sci. (2021) 17:3173–87. doi: 10.7150/ijbs.62556 PMC8375237 34421358

[51] Fedorenko A Lishko PV Kirichok Y. Mechanism of fatty-acid-dependent UCP1 uncoupling in brown fat mitochondria. Cell. (2012) 151:400–13. doi: 10.1016/j.cell.2012.09.010 PMC3782081 23063128

[52] Wang G Meyer JG Cai W Softic S Li ME Verdin E. Regulation of UCP1 and mitochondrial metabolism in brown adipose tissue by reversible succinylation. Mol Cell. (2019) 74:844–857.e7. doi: 10.1016/j.molcel.2019.03.021 31000437 PMC6525068

[53] Okamoto Y Higashiyama H Rong JX McVey MJ Kinoshita M Asano S. Comparison of mitochondrial and macrophage content between subcutaneous and visceral fat in db/db mice. Exp Mol Pathol. (2007) 83:73–83. doi: 10.1016/j.yexmp.2007.02.007 17434481

[54] Yau WW Yen PM. Thermogenesis in adipose tissue activated by thyroid hormone. Int J Mol Sci. (2020) 21:3020. doi: 10.3390/ijms21083020 32344721 PMC7215895

[55] Mills EL Pierce KA Jedrychowski MP Garrity R Winther S Vidoni S. Accumulation of succinate controls activation of adipose tissue thermogenesis. Nature. (2018) 560:102–6. doi: 10.1038/s41586-018-0353-2 PMC7045287 30022159

[56] Chouchani ET Kazak L Jedrychowski MP Lu GZ Erickson BK Szpyt J. Mitochondrial ROS regulate thermogenic energy expenditure and sulfenylation of UCP1. Nature. (2016) 532:112–6. doi: 10.1038/nature17399 PMC5549630 27027295

[57] Sanchez-Gurmaches J Tang Y Jespersen NZ Wallace M Martinez Calejman C Gujja S. Brown fat AKT2 is a cold-induced kinase that stimulates chREBP-mediated *de novo* lipogenesis to optimize fuel storage and thermogenesis. Cell Metab. (2018) 27:195–209.e6. doi: 10.1016/j.cmet.2017.10.008 29153407 PMC5762420

[58] Wei C Wang P Dong Q Ma X-H Lu M Qi S. ChREBP-regulated lipogenesis is not required for the thermogenesis of brown adipose tissue. Int J Obes (Lond). (2022) 46:1068–75. doi: 10.1038/s41366-022-01082-7 PMC8853070 35152269

[59] Xue K Wu D Wang Y Zhao Y Shen H Yao J. The mitochondrial calcium uniporter engages UCP1 to form a thermoporter that promotes thermogenesis. Cell Metab. (2022) 34:1325–1341.e6. doi: 10.1016/j.cmet.2022.07.011 35977541

[60] Yao L Heuser-Baker J Herlea-Pana O Zhang N Szweda LI Griffin TM. Deficiency in adipocyte chemokine receptor CXCR4 exacerbates obesity and compromises thermoregulatory responses of brown adipose tissue in a mouse model of diet-induced obesity. FASEB J. (2014) 28:4534–50. doi: 10.1096/fj.14-249797 PMC4202106 25016030

[61] Pazos P Lima L Tovar S González-Touceda D Diéguez C García MC. Divergent responses to thermogenic stimuli in BAT and subcutaneous adipose tissue from interleukin 18 and interleukin 18 receptor 1-deficient mice. Sci Rep. (2015) 5:17977. doi: 10.1038/srep17977 26656097 PMC4674707

[62] Harms M Seale P. Brown and beige fat: development, function and therapeutic potential. Nat Med. (2013) 19:1252–63. doi: 10.1038/nm.3361 24100998

[63] Nautiyal KM Dailey M Brito N Brito MNDA Harris RB Bartness TJ. Energetic responses to cold temperatures in rats lacking forebrain-caudal brain stem connections. Am J Physiol Regul Integr Comp Physiol. (2008) 295:R789–798. doi: 10.1152/ajpregu.90394.2008 PMC2536851 18635447

[64] Hsieh AC Carlson LD. Role of adrenaline and noradrenaline in chemical regulation of heat production. Am J Physiol. (1957) 190:243–6. doi: 10.1152/ajplegacy.1957.190.2.243 13458448

[65] Morrison SF Madden CJ Tupone D. Central neural regulation of brown adipose tissue thermogenesis and energy expenditure. Cell Metab. (2014) 19:741–56. doi: 10.1016/j.cmet.2014.02.007 PMC4016184 24630813

[66] Singh AK Aryal B Chaube B Rotllan N Varela L Horvath TL. Brown adipose tissue derived ANGPTL4 controls glucose and lipid metabolism and regulates thermogenesis. Mol Metab. (2018) 11:59–69. doi: 10.1016/j.molmet.2018.03.011 29627378 PMC6001401

[67] Min H-Y Hwang J Choi Y Jo Y-H. Overexpressing the hydroxycarboxylic acid receptor 1 in mouse brown adipose tissue restores glucose tolerance and insulin sensitivity in diet-induced obese mice. Am J Physiol Endocrinol Metab. (2022) 323:E231–41. doi: 10.1152/ajpendo.00084.2022 PMC9423771 35830691

[68] Qiang G Whang Kong H Gil V Liew CW. Transcription regulator TRIP-Br2 mediates ER stress-induced brown adipocytes dysfunction. Sci Rep. (2017) 7:40215. doi: 10.1038/srep40215 28067333 PMC5220316

[69] Underhill DM Gordon S Imhof BA Núñez G Bousso P. Élie Metchnikoff (1845-1916): celebrating 100 years of cellular immunology and beyond. Nat Rev Immunol. (2016) 16:651–6. doi: 10.1038/nri.2016.89 27477126

[70] Van den Bossche J O’Neill LA Menon D. Macrophage immunometabolism: where are we (Going)? Trends Immunol. (2017) 38:395–406. doi: 10.1016/j.it.2017.03.001 28396078

[71] Ryan DG Murphy MP Frezza C Prag HA Chouchani ET O’Neill LA. Coupling Krebs cycle metabolites to signalling in immunity and cancer. Nat Metab. (2019) 1:16–33. doi: 10.1038/s42255-018-0014-7 31032474 PMC6485344

[72] O’Neill LAJ Kishton RJ Rathmell J. A guide to immunometabolism for immunologists. Nat Rev Immunol. (2016) 16:553–65. doi: 10.1038/nri.2016.70 PMC5001910 27396447

[73] Zhang J Muri J Fitzgerald G Gorski T Gianni-Barrera R Masschelein E. Endothelial lactate controls muscle regeneration from ischemia by inducing M2-like macrophage polarization. Cell Metab. (2020) 31:1136–1153.e7. doi: 10.1016/j.cmet.2020.05.004 32492393 PMC7267778

[74] Colegio OR Chu N-Q Szabo AL Chu T Rhebergen AM Jairam V. Functional polarization of tumour-associated macrophages by tumour-derived lactic acid. Nature. (2014) 513:559–63. doi: 10.1038/nature13490 PMC4301845 25043024

[75] Zhang D Tang Z Huang H Zhou G Cui C Weng Y. Metabolic regulation of gene expression by histone lactylation. Nature. (2019) 574:575–80. doi: 10.1038/s41586-019-1678-1 PMC6818755 31645732

[76] van Kruijsdijk RCM van der Wall E Visseren FLJ. Obesity and cancer: the role of dysfunctional adipose tissue. Cancer Epidemiol Biomarkers Prev. (2009) 18:2569–78. doi: 10.1158/1055-9965.EPI-09-0372 19755644

[77] Xu X Grijalva A Skowronski A van Eijk M Serlie MJ Ferrante AW. Obesity activates a program of lysosomal-dependent lipid metabolism in adipose tissue macrophages independently of classic activation. Cell Metab. (2013) 18:816–30. doi: 10.1016/j.cmet.2013.11.001 PMC3939841 24315368

[78] Naylor C Petri WA. Leptin regulation of immune responses. Trends Mol Med. (2016) 22:88–98. doi: 10.1016/j.molmed.2015.12.001 26776093

[79] Silswal N Singh AK Aruna B Mukhopadhyay S Ghosh S Ehtesham NZ. Human resistin stimulates the pro-inflammatory cytokines TNF-alpha and IL-12 in macrophages by NF-kappaB-dependent pathway. Biochem Biophys Res Commun. (2005) 334:1092–101. doi: 10.1016/j.bbrc.2005.06.202 16039994

[80] Tilg H Moschen AR. Adipocytokines: mediators linking adipose tissue, inflammation and immunity. Nat Rev Immunol. (2006) 6:772–83. doi: 10.1038/nri1937 16998510

[81] West AP Shadel GS Ghosh S. Mitochondria in innate immune responses. Nat Rev Immunol. (2011) 11:389–402. doi: 10.1038/nri2975 21597473 PMC4281487

[82] Ma L Li W Zhang Y Qi L Zhao Q Li N. FLT4/VEGFR3 activates AMPK to coordinate glycometabolic reprogramming with autophagy and inflammasome activation for bacterial elimination. Autophagy. (2022) 18:1385–400. doi: 10.1080/15548627.2021.1985338 PMC9225223 34632918

[83] Ko MS Yun JY Baek I-J Jang JE Hwang JJ Lee SE. Mitophagy deficiency increases NLRP3 to induce brown fat dysfunction in mice. Autophagy. (2021) 17:1205–21. doi: 10.1080/15548627.2020.1753002 PMC8143238 32400277

[84] Liu L Inouye KE Allman WR Coleman AS Siddiqui S Hotamisligil GS. TACI-deficient macrophages protect mice against metaflammation and obesity-induced dysregulation of glucose homeostasis. Diabetes. (2018) 67:1589–603. doi: 10.2337/db17-1089 PMC6054430 29871859

[85] Zhou Q Wang Y Lu Z He C Li L You M. Cx43 acts as a mitochondrial calcium regulator that promotes obesity by inducing the polarization of macrophages in adipose tissue. Cell Signal. (2023) 105:110606. doi: 10.1016/j.cellsig.2023.110606 36681290

[86] Ding L Yuan X Yan J Huang Y Xu M Yang Z. Nrf2 exerts mixed inflammation and glucose metabolism regulatory effects on murine RAW264.7 macrophages. Int Immunopharmacol. (2019) 71:198–204. doi: 10.1016/j.intimp.2019.03.023 30913518

[87] Chang HR Kim HJ Xu X Ferrante AW. Macrophage and adipocyte IGF1 maintain adipose tissue homeostasis during metabolic stresses. Obes (Silver Spring). (2016) 24:172–83. doi: 10.1002/oby.21354 PMC4793714 26663512

[88] Ip WKE Hoshi N Shouval DS Snapper S Medzhitov R. Anti-inflammatory effect of IL-10 mediated by metabolic reprogramming of macrophages. Science. (2017) 356:513–9. doi: 10.1126/science.aal3535 PMC6260791 28473584

[89] Hirata A Maeda N Hiuge A Hibuse T Fujita K Okada T. Blockade of mineralocorticoid receptor reverses adipocyte dysfunction and insulin resistance in obese mice. Cardiovasc Res. (2009) 84:164–72. doi: 10.1093/cvr/cvp191 19505930

[90] Guo C Ricchiuti V Lian BQ Yao TM Coutinho P Romero JR. Mineralocorticoid receptor blockade reverses obesity-related changes in expression of adiponectin, peroxisome proliferator-activated receptor-gamma, and proinflammatory adipokines. Circulation. (2008) 117:2253–61. doi: 10.1161/CIRCULATIONAHA.107.748640 PMC2746647 18427128

[91] Chen L Zhang J Zou Y Wang F Li J Sun F. Kdm2a deficiency in macrophages enhances thermogenesis to protect mice against HFD-induced obesity by enhancing H3K36me2 at the Pparg locus. Cell Death Differ. (2021) 28:1880–99. doi: 10.1038/s41418-020-00714-7 PMC8185071 33462408

[92] Chen J Xu X Li Y Li F Zhang J Xu Q. Kdm6a suppresses the alternative activation of macrophages and impairs energy expenditure in obesity. Cell Death Differ. (2021) 28:1688–704. doi: 10.1038/s41418-020-00694-8 PMC8167088 33303977

[93] Engin AB. Adipocyte-macrophage cross-talk in obesity. Adv Exp Med Biol. (2017) 960:327–43. doi: 10.1007/978-3-319-48382-5_14 28585206

[94] Longo M Zatterale F Naderi J Parrillo L Formisano P Raciti GA. Adipose tissue dysfunction as determinant of obesity-associated metabolic complications. Int J Mol Sci. (2019) 20:2358. doi: 10.3390/ijms20092358 31085992 PMC6539070

[95] Hammarstedt A Gogg S Hedjazifar S Nerstedt A Smith U. Impaired adipogenesis and dysfunctional adipose tissue in human hypertrophic obesity. Physiol Rev. (2018) 98:1911–41. doi: 10.1152/physrev.00034.2017 30067159

[96] Michailidou Z Gomez-Salazar M Alexaki VI. Innate immune cells in the adipose tissue in health and metabolic disease. J Innate Immun. (2022) 14:4–30. doi: 10.1159/000515117 33849008 PMC8787575

[97] Xu H Barnes GT Yang Q Tan G Yang D Chou CJ. Chronic inflammation in fat plays a crucial role in the development of obesity-related insulin resistance. J Clin Invest. (2003) 112:1821–30. doi: 10.1172/JCI19451 PMC296998 14679177

[98] Weisberg SP McCann D Desai M Rosenbaum M Leibel RL Ferrante AW. Obesity is associated with macrophage accumulation in adipose tissue. J Clin Invest. (2003) 112:1796–808. doi: 10.1172/JCI19246 PMC296995 14679176

[99] Nedergaard J Bengtsson T Cannon B. New powers of brown fat: fighting the metabolic syndrome. Cell Metab. (2011) 13:238–40. doi: 10.1016/j.cmet.2011.02.009 21356513

[100] Bienboire-Frosini C Wang D Marcet-Rius M Villanueva-García D Gazzano A Domínguez-Oliva A. The role of brown adipose tissue and energy metabolism in mammalian thermoregulation during the perinatal period. Anim (Basel). (2023) 13:2173. doi: 10.3390/ani13132173 PMC10339909 37443971

[101] Wolf Y Boura-Halfon S Cortese N Haimon Z Sar Shalom H Kuperman Y. Brown-adipose-tissue macrophages control tissue innervation and homeostatic energy expenditure. Nat Immunol. (2017) 18:665–74. doi: 10.1038/ni.3746 PMC5438596 28459435

[102] Luo Y Liu B Yang X Ma X Zhang X Bragin DE. Myeloid adrenergic signaling *via* CaMKII forms a feedforward loop of catecholamine biosynthesis. J Mol Cell Biol. (2017) 9:422–34. doi: 10.1093/jmcb/mjx046 PMC5907839 29087480

[103] Wang Y-N Tang Y He Z Ma H Wang L Liu Y. Slit3 secreted from M2-like macrophages increases sympathetic activity and thermogenesis in adipose tissue. Nat Metab. (2021) 3:1536–51. doi: 10.1038/s42255-021-00482-9 34782792

[104] Fischer K Ruiz HH Jhun K Finan B Oberlin DJ van der Heide V. Alternatively activated macrophages do not synthesize catecholamines or contribute to adipose tissue adaptive thermogenesis. Nat Med. (2017) 23:623–30. doi: 10.1038/nm.4316 PMC5420449 28414329

[105] Wang F Xiao F Du L Niu Y Yin H Zhou Z. Activation of GCN2 in macrophages promotes white adipose tissue browning and lipolysis under leucine deprivation. FASEB J. (2021) 35:e21652. doi: 10.1096/fj.202100061RR 34004054

[106] Nisoli E Tonello C Briscini L Carruba MO. Inducible nitric oxide synthase in rat brown adipocytes: implications for blood flow to brown adipose tissue. Endocrinology. (1997) 138:676–82. doi: 10.1210/endo.138.2.4956 9003002

[107] Pirzgalska RM Seixas E Seidman JS Link VM Sánchez NM Mahú I. Sympathetic neuron-associated macrophages contribute to obesity by importing and metabolizing norepinephrine. Nat Med. (2017) 23:1309–18. doi: 10.1038/nm.4422 PMC7104364 29035364

[108] Zhang H Zhang Sy Jiang C Li Y Xu G Xu Mj. Intermedin/adrenomedullin 2 polypeptide promotes adipose tissue browning and reduces high-fat diet-induced obesity and insulin resistance in mice. Int J Obes (2005). (2016) 40(5):852–60. doi: 10.1038/ijo.2016 26786353

[109] Lv Y Zhang S-Y Liang X Zhang H Xu Z Liu B. Adrenomedullin 2 enhances beiging in white adipose tissue directly in an adipocyte-autonomous manner and indirectly through activation of M2 macrophages. J Biol Chem. (2016) 291:23390–402. doi: 10.1074/jbc.M116.735563 PMC5095396 27621315

[110] Qiu Y Nguyen KD Odegaard JI Cui X Tian X Locksley RM. Eosinophils and type 2 cytokine signaling in macrophages orchestrate development of functional beige fat. Cell. (2014) 157:1292–308. doi: 10.1016/j.cell.2014.03.066 PMC4129510 24906148

[111] Li L Ma L Zhao Z Luo S Gong B Li J. IL-25-induced shifts in macrophage polarization promote development of beige fat and improve metabolic homeostasis in mice. PloS Biol. (2021) 19:e3001348. doi: 10.1371/journal.pbio.3001348 34351905 PMC8341513

[112] Knights AJ Liu S Ma Y Nudell VS Perkey E Sorensen MJ. Acetylcholine-synthesizing macrophages in subcutaneous fat are regulated by β2 -adrenergic signaling. EMBO J. (2021) 40:e106061. doi: 10.15252/embj.2020106061 34459015 PMC8672283

[113] Feng J Li L Ou Z Li Q Gong B Zhao Z. IL-25 stimulates M2 macrophage polarization and thereby promotes mitochondrial respiratory capacity and lipolysis in adipose tissues against obesity. Cell Mol Immunol. (2018) 15:493–505. doi: 10.1038/cmi.2016.71 28194019 PMC6068125

[114] Brestoff JR Kim BS Saenz SA Stine RR Monticelli LA Sonnenberg GF. Group 2 innate lymphoid cells promote beiging of white adipose tissue and limit obesity. Nature. (2015) 519:242–6. doi: 10.1038/nature14115 PMC4447235 25533952

[115] Stanford KI Middelbeek RJW Townsend KL Lee M-Y Takahashi H So K. A novel role for subcutaneous adipose tissue in exercise-induced improvements in glucose homeostasis. Diabetes. (2015) 64:2002–14. doi: 10.2337/db14-0704 PMC4439563 25605808

[116] During MJ Liu X Huang W Magee D Slater A McMurphy T. Adipose VEGF links the white-to-brown fat switch with environmental, genetic, and pharmacological stimuli in male mice. Endocrinology. (2015) 156:2059–73. doi: 10.1210/en.2014-1905 PMC4430610 25763639

[117] Xue Y Petrovic N Cao R Larsson O Lim S Chen S. Hypoxia-independent angiogenesis in adipose tissues during cold acclimation. Cell Metab. (2009) 9:99–109. doi: 10.1016/j.cmet.2008.11.009 19117550

[118] Keuper M Sachs S Walheim E Berti L Raedle B Tews D. Activated macrophages control human adipocyte mitochondrial bioenergetics *via* secreted factors. Mol Metab. (2017) 6:1226–39. doi: 10.1016/j.molmet.2017.07.008 PMC5641636 29031722

[119] Okla M Zaher W Alfayez M Chung S. Inhibitory effects of toll-like receptor 4, NLRP3 inflammasome, and interleukin-1β on white adipocyte browning. Inflammation. (2018) 41:626–42. doi: 10.1007/s10753-017-0718-y PMC6066287 29264745

[120] Crespo M Nikolic I Mora A Rodríguez E Leiva-Vega L Pintor-Chocano A. Myeloid p38 activation maintains macrophage-liver crosstalk and BAT thermogenesis through IL-12-FGF21 axis. Hepatology. (2023) 77:874–87. doi: 10.1002/hep.32581 PMC9936978 35592906

[121] Moreno-Navarrete JM Ortega F Gómez-Serrano M García-Santos E Ricart W Tinahones F. The MRC1/CD68 ratio is positively associated with adipose tissue lipogenesis and with muscle mitochondrial gene expression in humans. PloS One. (2013) 8:e70810. doi: 10.1371/journal.pone.0070810 23951013 PMC3741275

[122] Hahn WS Kuzmicic J Burrill JS Donoghue MA Foncea R Jensen MD. Proinflammatory cytokines differentially regulate adipocyte mitochondrial metabolism, oxidative stress, and dynamics. Am J Physiol Endocrinol Metab. (2014) 306:E1033–1045. doi: 10.1152/ajpendo.00422.2013 PMC4010657 24595304

[123] Liu P Huang G Cao Z Xie Q Wei T Huang C. Haematopoietic TLR4 deletion attenuates perivascular brown adipose tissue inflammation in atherosclerotic mice. Biochim Biophys Acta Mol Cell Biol Lipids. (2017) 1862:946–57. doi: 10.1016/j.bbalip.2017.05.012 28579235

[124] Brestoff JR Wilen CB Moley JR Li Y Zou W Malvin NP. Intercellular mitochondria transfer to macrophages regulates white adipose tissue homeostasis and is impaired in obesity. Cell Metab. (2021) 33:270–282.e8. doi: 10.1016/j.cmet.2020.11.008 33278339 PMC7858234

[125] Hotamisligil GS. Inflammation, metaflammation and immunometabolic disorders. Nature. (2017) 542:177–85. doi: 10.1038/nature21363 28179656

[126] Rosina M Ceci V Turchi R Chuan L Borcherding N Sciarretta F. Ejection of damaged mitochondria and their removal by macrophages ensure efficient thermogenesis in brown adipose tissue. Cell Metab. (2022) 34:533–548.e12. doi: 10.1016/j.cmet.2022.02.016 35305295 PMC9039922

[127] Aquilano K Zhou B Brestoff JR Lettieri-Barbato D. Multifaceted mitochondrial quality control in brown adipose tissue. Trends Cell Biol. (2023) 33:517–29. doi: 10.1016/j.tcb.2022.09.008 PMC11657393 36272883

[128] Xu H Hertzel AV Steen KA Bernlohr DA. Loss of fatty acid binding protein 4/aP2 reduces macrophage inflammation through activation of SIRT3. Mol Endocrinol. (2016) 30:325–34. doi: 10.1210/me.2015-1301 PMC4771695 26789108

[129] Steen KA Xu H Bernlohr DA. FABP4/aP2 regulates macrophage redox signaling and inflammasome activation *via* control of UCP2. Mol Cell Biol. (2017) 37:e00282–16. doi: 10.1128/MCB.00282-16 PMC5214853 27795298

[130] Seo SH Jo S-M Truong TTM Zhang G Kim D-S Lee M. Peanut sprout rich in p-coumaric acid ameliorates obesity and lipopolysaccharide-induced inflammation and the inhibition of browning in adipocytes *via* mitochondrial activation. Food Funct. (2021) 12:5361–74. doi: 10.1039/D1FO00342A 33982705

[131] Suganami T Tanimoto-Koyama K Nishida J Itoh M Yuan X Mizuarai S. Role of the Toll-like receptor 4/NF-kappaB pathway in saturated fatty acid-induced inflammatory changes in the interaction between adipocytes and macrophages. Arterioscler Thromb Vasc Biol. (2007) 27:84–91. doi: 10.1161/01.ATV.0000251608.09329.9a 17082484

[132] Bjursell M Admyre T Göransson M Marley AE Smith DM Oscarsson J. Improved glucose control and reduced body fat mass in free fatty acid receptor 2-deficient mice fed a high-fat diet. Am J Physiol Endocrinol Metab. (2011) 300:E211–220. doi: 10.1152/ajpendo.00229.2010 20959533

[133] Odegaard JI Chawla A. Alternative macrophage activation and metabolism. Annu Rev Pathol. (2011) 6:275–97. doi: 10.1146/annurev-pathol-011110-130138 PMC3381938 21034223

[134] Olefsky JM Glass CK. Macrophages, inflammation, and insulin resistance. Annu Rev Physiol. (2010) 72:219–46. doi: 10.1146/annurev-physiol-021909-135846 20148674

[135] Odegaard JI Ricardo-Gonzalez RR Goforth MH Morel CR Subramanian V Mukundan L. Macrophage-specific PPARgamma controls alternative activation and improves insulin resistance. Nature. (2007) 447:1116–20. doi: 10.1038/nature05894 PMC2587297 17515919

[136] Sajic T Hainard A Scherl A Wohlwend A Negro F Sanchez J-C. STAT6 promotes bi-directional modulation of PKM2 in liver and adipose inflammatory cells in rosiglitazone-treated mice. Sci Rep. (2013) 3:2350. doi: 10.1038/srep02350 23917405 PMC3734444

[137] Chawla A. Control of macrophage activation and function by PPARs. Circ Res. (2010) 106:1559–69. doi: 10.1161/CIRCRESAHA.110.216523 PMC2897247 20508200

[138] Boutens L Hooiveld GJ Dhingra S Cramer RA Netea MG Stienstra R. Unique metabolic activation of adipose tissue macrophages in obesity promotes inflammatory responses. Diabetologia. (2018) 61:942–53. doi: 10.1007/s00125-017-4526-6 PMC6448980 29333574

[139] Surendar J Frohberger SJ Karunakaran I Schmitt V Stamminger W Neumann A-L. Adiponectin limits IFN-γ and IL-17 producing CD4 T cells in obesity by restraining cell intrinsic glycolysis. Front Immunol. (2019) 10:2555. doi: 10.3389/fimmu.2019.02555 31736971 PMC6828851

[140] Jaitin DA Adlung L Thaiss CA Weiner A Li B Descamps H. Lipid-associated macrophages control metabolic homeostasis in a trem2-dependent manner. Cell. (2019) 178:686–698.e14. doi: 10.1016/j.cell.2019.05.054 31257031 PMC7068689

[141] Snodgrass RG Boß M Zezina E Weigert A Dehne N Fleming I. Hypoxia potentiates palmitate-induced pro-inflammatory activation of primary human macrophages. J Biol Chem. (2016) 291:413–24. doi: 10.1074/jbc.M115.686709 PMC4697177 26578520

[142] Corcoran SE O’Neill LAJ. HIF1α and metabolic reprogramming in inflammation. J Clin Invest. (2016) 126:3699–707. doi: 10.1172/JCI84431 PMC5096812 27571407

[143] Sharma M Boytard L Hadi T Koelwyn G Simon R Ouimet M. Enhanced glycolysis and HIF-1α activation in adipose tissue macrophages sustains local and systemic interleukin-1β production in obesity. Sci Rep. (2020) 10:5555. doi: 10.1038/s41598-020-62272-9 32221369 PMC7101445

[144] Tannahill GM Curtis AM Adamik J Palsson-McDermott EM McGettrick AF Goel G. Succinate is an inflammatory signal that induces IL-1β through HIF-1α. Nature. (2013) 496:238–42. doi: 10.1038/nature11986 PMC4031686 23535595

[145] Goto T Naknukool S Yoshitake R Hanafusa Y Tokiwa S Li Y. Proinflammatory cytokine interleukin-1β suppresses cold-induced thermogenesis in adipocytes. Cytokine. (2016) 77:107–14. doi: 10.1016/j.cyto.2015.11.001 26556104

[146] Satoh T Kidoya H Naito H Yamamoto M Takemura N Nakagawa K. Critical role of Trib1 in differentiation of tissue-resident M2-like macrophages. Nature. (2013) 495:524–8. doi: 10.1038/nature11930 23515163

[147] Valdearcos M Robblee MM Benjamin DI Nomura DK Xu AW Koliwad SK. Microglia dictate the impact of saturated fat consumption on hypothalamic inflammation and neuronal function. Cell Rep. (2014) 9:2124–38. doi: 10.1016/j.celrep.2014.11.018 PMC4617309 25497089

[148] Valdearcos M Douglass JD Robblee MM Dorfman MD Stifler DR Bennett ML. Microglial inflammatory signaling orchestrates the hypothalamic immune response to dietary excess and mediates obesity susceptibility. Cell Metab. (2018) 27:1356. doi: 10.1016/j.cmet.2018.04.019 29874568

[149] Akifusa S Kamio N Shimazaki Y Yamaguchi N Nishihara T Yamashita Y. Globular adiponectin-induced RAW 264 apoptosis is regulated by a reactive oxygen species-dependent pathway involving Bcl-2. Free Radic Biol Med. (2009) 46:1308–16. doi: 10.1016/j.freeradbiomed.2009.02.014 19250964

[150] Zhang T Zhang Z Xia T Liu C Sun C. circNrxn2 Promoted WAT Browning *via* Sponging miR-103 to Relieve Its Inhibition of FGF10 in HFD Mice. Mol Ther Nucleic Acids. (2019) 17:551–62. doi: 10.1016/j.omtn.2019.06.019 PMC6661467 31362242

[151] Campderrós L Moure R Cairó M Gavaldà-Navarro A Quesada-López T Cereijo R. Brown adipocytes secrete GDF15 in response to thermogenic activation. Obes (Silver Spring). (2019) 27:1606–16. doi: 10.1002/oby.22584 31411815

[152] Hui X Gu P Zhang J Nie T Pan Y Wu D. Adiponectin enhances cold-induced browning of subcutaneous adipose tissue *via* promoting M2 macrophage proliferation. Cell Metab. (2015) 22:279–90. doi: 10.1016/j.cmet.2015.06.004 26166748

[153] Cereijo R Gavaldà-Navarro A Cairó M Quesada-López T Villarroya J Morón-Ros S. CXCL14, a brown adipokine that mediates brown-fat-to-macrophage communication in thermogenic adaptation. Cell Metab. (2018) 28:750–763.e6. doi: 10.1016/j.cmet.2018.07.015 30122557

